# Breathtaking dreams: reduced REM phenotype in REM-related sleep apnea

**DOI:** 10.1007/s11325-024-03236-5

**Published:** 2025-01-22

**Authors:** Luca Cerina, Pedro Fonseca, Gabriele B. Papini, Rik Vullings, Sebastiaan Overeem

**Affiliations:** 1 Electrical Engineering, Eindhoven University of Technology, Eindhoven, The Netherlands; 2 Sleep & Respiratory Care, Philips, Eindhoven, The Netherlands; 3 Hospital Patient Monitoring, Philips, Eindhoven, The Netherlands; 4 Center for Sleep Medicine, Kempenhaeghe, Heeze, The Netherlands

**Keywords:** REM-related sleep apnea, Obstructive sleep apnea, REM sleep, Rapid eye movement, Sleep-disordered breathing

## Abstract

**Purpose:**

The expression of the respiratory events in OSA is influenced by different mechanisms. In particular, REM sleep can highly increase the occurrence of events in a subset of OSA patients, a condition dubbed REM-OSA (often defined as an AHI 2 times higher in REM than NREM sleep). However, a proper characterization of REM-OSA and its pathological sequelae is still inadequate, partly because of limitations in the current definitions.

**Methods:**

We propose a new interpretation of the REM-OSA definition, extending it from a AHI-ratio to a two-dimensional space, considering both time and events ratios in REM over NREM separately. Within this space, we analyzed current definitions of REM-OSA in three large clinical dataset and identified the underlying sources of heterogeneity.

**Results:**

We observed that REM-OSA and REM-independent-OSA subgroups exist. Some subgroups exhibited abnormal REM characteristics (e.g., REM-OSA with reduced time in REM). Others had OSA features that are intermediate between REM-independent-OSA participants and those with a clear disproportion of REM events.

**Conclusion:**

We found that a time and events’ ratio of REM and NREM allow a more precise characterization of REM-OSA subgroups. Our new interpretation can be used to bolster new research into REM-OSA pathophysiological mechanisms.

**Supplementary Information:**

The online version contains supplementary material available at 10.1007/s11325-024-03236-5.

## Introduction

SDB and specifically OSA, is a highly prevalent disease characterized by breathing interruptions during sleep. SDB pathophysiology depends on many demographic (e.g., age and sex), bodily (obesity, cranial morphology), and functional (e.g., sleep posture) factors. For example, the presence of different sleep stages, and of REM sleep in particular, can significantly influence the occurrence of respiratory events. OSA severity is defined by the frequency of these events, the AHI, or the total number of events over the time spent asleep. Alongside general muscle atonia (which prevents dream acting), REM sleep also affects respiration: decreased genioglossus activity increases upper airway collapsibility, while altered chemosensitivity to blood gases lowers respiratory drive from the diaphragm [1]. The combined effect leads to a higher risk of respiratory events, both obstructive and central.

When the increase of respiratory events during REM is disproportionate compared to non-REM (NREM) events, the manifestation of OSA, its *phenotype*, is referred to as REM-dominant or REM-related OSA (REM-OSA in the remainder of this paper). REM-OSA has been associated with higher health risks compared to non-stage specific OSA, including hypertension [2] cardiovascular events [3], worse sleep-related quality of life [4], and increased insulin resistance [5].

The definition of a relevant disproportion of events between REM and NREM is still subject of debate. In one of the earliest works, Haba-Rubio et al*.* [6] proposed as a threshold for REM-OSA a ratio of $AH{I}_{REM}/AH{I}_{NREM}>2$, and this remains the most common rule used in literature. REM-OSA, according to this definition, is found in 37% of the OSA population, has a general association with milder OSA severity and female sex, but also with a heterogeneous manifestation of objective and subjective symptoms [7].

Subsequent research criticized this formulation, observing how a ratio of ratios of tightly interdependent mechanisms may not be specific enough and can be easily wrongly interpreted. A recent review from Bonsignore et al*.* provides a comprehensive overview of the literature [8]. Some authors proposed alternative and stricter rules to increase the specificity when defining the phenotype: Conwell et al*.* extended the formula with with the requirement that the AHI during NREM should be low ($<15$ events/hr [9], *Predominant* REM-OSA in [8]), while Mokhlesi et al*.* proposed that virtually no events should happen in NREM ($AH{I}_{NREM}$ below 5 events/hr) and that at least 30 min of REM sleep should be recorded ([7], *Isolated* REM-OSA in [8]). All rules concern people with an overall $AHI>5$. In the remainder of the paper, the REM-OSA term without qualifiers will indicate the original formulation, while we will borrow the terminology from [8] to indicate *predominant* [9] and *isolated* [7] REM-OSA. In this paper we will refer to REM-independent-OSA as non REM-OSA with respect to existing definitions, which do not allow the separation of REM-independent-OSA as NREM-prevalent OSA or a truly sleep stage-neutral OSA.

Unfortunately, increased specificity comes with the unintended effect of conflating the remaining population. The now "REM-independent-OSA" group may contain people with truly sleep stage independent events and others whose events during REM remain a hypothetically important pathological driver, but which do not count towards the calculation of the AHI ratios (for example, if they precede a long bout of wakefulness). We propose here a more systematic approach to the definition of REM-related phenotypes.

Previous research observed the dependency of REM-OSA on the natural ratio between REM and NREM time (with the average time in REM being around 20% of total sleep time, and most commonly below 25% in adults [10]) and how that may influence the AHI ratio [7, 11–13]. We aim to understand better how the probability of REM events might be intrinsically conditioned by the time spent in REM. We therefore deconstructed the $AH{I}_{ratio}$ in two components: an REM/NREM *time* ratio and a REM/NREM *events* ratio. The main goal of this analysis is to understand if the original formulation is too broad, thereby validating more conservative thresholds such as those defined for *predominant*- and *isolated* REM-OSA, or if there is an underlying heterogeneity that requires further attention. For sake of conciseness, we will focus on REM-OSA as the most general definition, and *isolated* REM-OSA as the most strict, but our approach remains valid also for *predominant* REM-OSA. With this two-dimensional representation of $AH{I}_{ratio}$, we aimed to characterize the source of heterogeneity in REM-OSA and *isolated* REM-OSA groups, their consistency across different clinical datasets, and the possible emergence of a new REM-OSA phenotype with a marked REM reduction.

## Methods

### Dataset and data selection

This study made use of de-identified PSGs from multiple resources. The SHHS and MESA (specifically MESA-sleep, here referred as MESA for brevity) from the NSRR [14] and the SOMNIA dataset from the Center for Sleep Medicine Kempenhaeghe (Heeze, the Netherlands) [15]. The three datasets add up to a total of 8566 unique PSG recordings and each of them was designed for different purposes. SHHS was a multi-site, longitudinal cohort study aimed to investigate the effects of SDB as a risk factor for cardiovascular diseases. MESA was a multi-site, longitudinal cohort study with the objective to understand how SDB and, more generally, sleep relate to the development and progression of atherosclerosis across gender and ethnic groups. SOMNIA is an ongoing study designed to facilitate research on unobtrusive monitoring of sleep and sleep disorders with patients undergoing PSG for suspicion of sleep disorders (including, but not limited to OSA) as third-line referral. For all the datasets, we employed the data available from the first PSG visit. We do not have information on how many participants were PSG-naive. All procedures performed in studies involving human participants were in accordance with the ethical standards of the institutional and/or national research committee (SOMNIA: Maxima Medical Center, Eindhoven, the Netherlands, File no: N16.074, SHHS/MESA: refer to original publications) and with the 1964 Helsinki declaration and its later amendments or comparable ethical standards. Informed consent was obtained from all individual participants included in the study.

Since different datasets were acquired in different years, with different equipment, and different sleep scoring guidelines (e.g.,AASM or older R&K rules) the exact interpretation of sleep stages, arousals and respiratory events may differ. To avoid incongruities in the analysis caused by these factors, we opted to re-score and harmonize all recordings using the Somnolyzer 4.1 software (Philips, Monroeville, PA, USA) [16]. The software is designed to follow current AASM guidelines v2.6 [17] (and has been validated in multiple cohorts, achieving, in comparison with manual scoring, an accuracy across sleep stages of 80.7% and a Cohen’s kappa of 0.739 [16]). It automatically scores sleep stages and cortical arousals based on the available EEG, EOG and chin EMG channels, and respiratory events (including RERA) using the oronasal thermal or pressure airflow, thoracic and abdominal RIP belts, and oxygen saturation from finger oximetry.

To uniform our analyses with existing literature, we performed a basic selection of recordings according to the following rules: AHI > = 5, at least 4 h of recorded data, and a minimum of 30 min in REM sleep (as in [7]). This resulted in a total of 5226, 1267, and 299 recordings from SHHS, MESA, and SOMNIA, respectively, for a combined total of 6792 recordings that were used for further analyses.

Table 1 reports demographic characteristics alongside basic sleep and OSA metrics for all included data. The table also indicates how many participants would be classified as REM-OSA or *Isolated* REM-OSA according to previously mentioned formulations [6, 7]. We should note some differences between the datasets we employed in terms of demographics (age, sex, and ethnicity), sleep and OSA metrics, as well as data collection methodologies. We unified the results of all three datasets in Sect. 3 to improve the sample size and the generalizability of our observations. The results obtained in the separate analysis of each dataset can be found in the supplemental materials.

**Table 1:** Demographics of the datasets employed. Each column indicates the number of recordings from each dataset after application of the initial selection: AHI > = 5, at least 4 h of data, and a minimum of 30 min in REM sleep

		SHHS	MESA	SOMNIA
Recordings [#]		5226	1267	299
Demographics				
Sex	[F/M]	2741 / 2485	708 / 559	89 / 210
Age	[years]	62.7 ± 11.1	69.0 ± 9.0	52.9 ± 12.3
Body-mass index (BMI)	[kg/m^2^]	28.2 ± 5.0	NA	28.3 ± 4.7
Sleep Metrics				
Total sleep time (TST)	[mins]	378.9 ± 55.7	378.8 ± 67.4	402.6 ± 59.9
Time in REM	[%]	19.6 ± 5.2	18.1 ± 5.6	18.8 ± 4.9
OSA Metrics AHI	[events/hr]	34.0 ± 19.5	35.2 ± 20.0	24.4 ± 17.0
- Apnea Index	[events/hr]	4.6 ± 8.7	3.8 ± 7.2	4.8 ± 9.5
- Hypopnea Index	[events/hr]	29.4 ± 15.8	31.4 ± 17.4	19.7 ± 12.9
Fraction hypopneas	[%]	89.8 ± 13.6	91.6 ± 12.1	85.4 ± 18.2
Ratio AHI REM/NREM	[-]	3.4 ± 5.1	3.2 ± 4.4	2.3 ± 3.7
REM-OSA^*∗*^	[F/M]	1903 / 949	439 / 193	43 / 48
*Isolated* REM-OSA^*†*^	[F/M]	269 / 70	56 / 16	17 / 15

^*^According to Haba-Rubio et al*.* definition [6]

^†^According to Mokhlesi et al*.* definition [7]

### REM-related AHI ratio

To explore the interaction of OSA with REM and NREM sleep, we start from the REM-OSA formulation of Haba-Rubio et al*.*:

1 $$AH{I}_{ratio}>2\doteq \frac{AH{I}_{REM}}{AH{I}_{NREM}}>2$$

This inequality can be true in different ways: with a proper $>$ 2:1 ratio (and trivial multiples e.g., 10:5, 20:10, etc..), with a relatively large numerator (e.g., 4:1), or with a relatively small denominator (e.g., 2:0.5). The $AH{I}_{ratio}$ can be thus represented in a two-dimensional space, that becomes clear as we decompose the AHI factors as:

2 $$\frac{\# {\mathrm{events}}_{REM}}{tim{e}_{REM}}/\frac{\# {\mathrm{events}}_{NREM}}{tim{e}_{NREM}}>2$$

The division can be rewritten as a multiplication, where we can separately factor time and event ratios:

3 $$\begin{array}{cc}(\frac{tim{e}_{REM}}{tim{e}_{NREM}}{)}^{-1}& \times \frac{\# {\mathrm{events}}_{REM}}{\# {\mathrm{events}}_{NREM}}>2\\ {T}_{ratio}& \times E{v}_{ratio}>2\end{array}$$

**Fig. 1:**
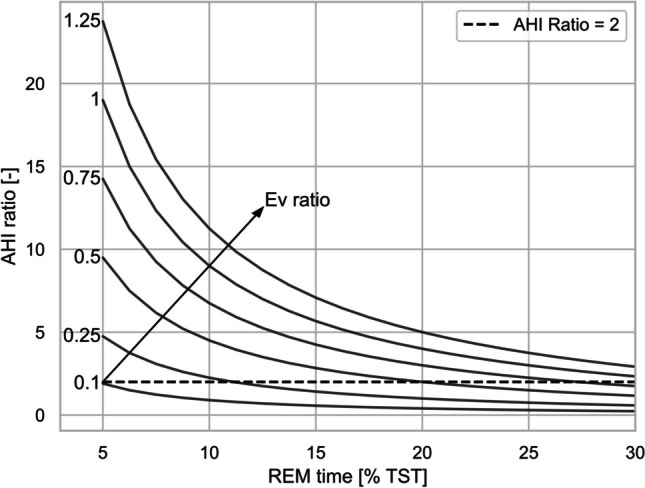
*AHI*_*ration*_ as a function of time in REM and growing *Ev*_*ratio*_. The dashed line indicates the cutoff threshold of *AHI*_*ration*_ =2

Equation 1 and Eq. 3 are mathematically equivalent, therefore the number of participants classified as REM-OSA using Eq. 3 are the same presented in Table 1.1 using the definition from [6]. We can then consider the REM/NREM events ratio ($E{v}_{ratio}$ onwards) to be positive and theoretically without a defined upper boundary (in practice we measured a maximum at 67 and a 99th percentile around 17 in our dataset), while the REM/NREM time ratio (${T}_{ratio}$ onwards) is limited by the binary nature of REM and NREM sleep (at least in our scope of analysis) and the overall time asleep. If we consider an average time in REM of $\approx 20\mathrm{\%}$ of TST (or 1:4 ${T}_{ratio}$) then an $E{v}_{ratio}$ of 1:2 is sufficient for the inequality of Eq. 3 to be true, or in other words: every event in REM weighs as much as two events in NREM. Consequently, if the time in REM increases (e.g., to 25%) then the relative weight of REM events contributing to the threshold of REM-OSA will decrease. Vice versa, less time in REM will increase the relative weight of REM events. A graphical representation of the relationship between time in REM sleep and $E{v}_{ratio}$ is provided in Fig. 1

Naturally, the same two-dimensional space exists using $AH{I}_{REM}$ and $AH{I}_{NREM}$. What could make the two new ratios useful is the ability to (partially) decouple the relative time spent in different sleep stages and the occurrence of respiratory events.

### Feature selection

In the new two-dimensional space based on ${T}_{ratio}$ and $E{v}_{ratio}$ we can explore not only if, but also why a certain person may be classified as REM-OSA or not, and assess the validity of existing membership rules. To test our hypotheses, we calculated a variety of sleep and OSA-related features and compared them between various REM-independent- and REM-OSA subgroups. In an ideal scenario, REM-independent- and REM-OSA phenotypes should exhibit good clustering properties: highly homogeneous characteristics inside the same group, and distinctive characteristics between groups. We considered different polysomnographic features covering sleep architecture and quality (e.g., total sleep time, sleep efficiency, time in REM), sleep disturbances (e.g., WASO, frequency of arousals and awakenings), and OSA-related (e.g., AHI, duration of events, patterns of oxygen desaturations). Supine position is also a relevant risk factor for respiratory events, we included the time in supine position as percentage of total sleep time, and the time supine during REM sleep as percentage of total REM time to assess potential differences between groups. We opted to not report the $AH{I}_{ratio}$ of supine and non-supine position (commonly used to define positional sleep apnea), as it has similar caveats of REM/NREM’s $AH{I}_{ratio}$. A proper in-depth analysis is outside the main focus of this paper. Among more recent metrics, we included the hypoxic burden [18] and the WASO of wake bouts longer than 5 min [19], which relate to cardiovascular risk and co-morbid insomnia, respectively. With respect to subjective symptoms, only the ESS was available across all datasets. Other metrics for insomnia, anxiety, and depression and other quality of life and sleep metrics were present in the datasets, but quantified differently, so that direct comparisons were difficult and outside the main topic of this work.

Given the importance of REM architecture in our analysis, we introduced a new metric expressing the difference between WASO and REM time (WASO-REM). Broadly speaking, a positive WASO-REM suggests that REM sleep may be lost to wakefulness. Vice-versa, a negative value means more REM sleep than wakefulness. Ultimately, we selected 23 unique polysomnographic variables, plus demographic and anthropometric variables: sex, age and BMI (not available in MESA dataset).

### Statistical analysis

We tested statistical differences in previously defined REM-independent- and REM-OSA groups, and extended them with potential additional subgroups based on ${T}_{ratio}$ and $E{v}_{ratio}$. We focused on REM-OSA and *isolated* REM-OSA as the loosest and strictest definitions, respectively, and left *predominant* REM-OSA out of the main discussion.

As a first approximation, we used the general REM-OSA criterion to divide the sample in REM-OSA and REM-independent-OSA groups using, as explained, the cutoff $AH{I}_{ratio}>2$. Then, we annotated the REM-OSA participants as *HighEvr* if $E{v}_{ratio}$ was above 0.5 and *LowEvr* otherwise. The REM-independent-OSA participants were not divided based on this criterion, since less than 1% of those recordings had an $E{v}_{ratio}>0.5$.

**Fig. 2:**
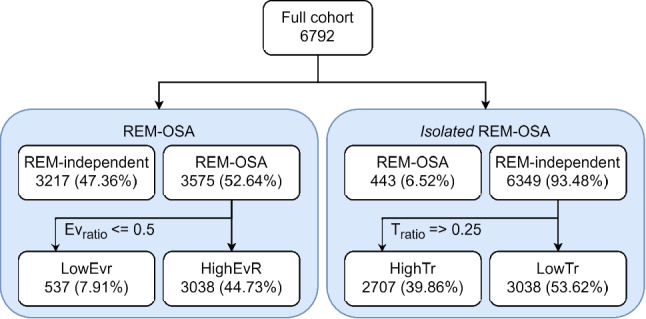
Separation of the whole sample in subgroups according to rules from Haba-Rubio et al*.* (REM-OSA), Mokhlesi et al*.* (Isolated REM-OSA) and *T*_*ratio*_ and *Ev*_*ratio*_ splits

In a second analysis, we used the *isolated* REM-OSA ($AH{I}_{ratio}>2$ $AH{I}_{NREM}<5$ and at least 30 min of REM sleep) criteria to divide our cohort in REM-OSA and REM-independent-OSA groups. We then used a ${T}_{ratio}$ threshold of 0.25 (meaning 20% of total sleep time in REM) to separate the (hypothetically) REM-independent-OSA group in two sub-groups: *LowTr* if ${T}_{ratio}$ was below 0.25 (time in NREM more than 80% of total sleep time), *HighTr* otherwise. The REM-OSA group was not separated further as it is small enough, covering less than 7% of our cohort. Figure 2 shows the different subgroups in our sample, with relevant percentages. Details and methodological consideration on the appropriateness of thresholds used to separate groups are available in Appendix 1.6.

We tested statistical differences between subgroups using Kruskal–Wallis test and post-hoc two-sided Dunn’s test. Significance level was set to $\alpha =0.01$. To evaluate differences in proportions (e.g., regarding the prevalence of females) we employed the two proportion Z-test. We controlled for multiple tests false discovery rate with the Benjamini–Hochberg procedure (10% error rate).

It is worthwhile to anticipate that almost all observed comparisons were significant with $p<.001$, even after controlling for false discovery; this is possibly an artifact caused by the large sample size [20]. Therefore, we included effect size measures to help interpret the results (more on the subject in [21]). We measured the Kruskal–Wallis’ effect size with ${\eta }^{2}$ (unbounded, marking comparison with ${\eta }^{2}>0.06$ as interesting) and two groups effect size with Cliff’s delta $d$ (bounded [-1:1]). We included also an analysis on the effect of biological sex, following current guidelines and given the expected high prevalence of females in REM-OSA populations (according to previous literature). For each identified subgroup, we calculated the Cliff’s delta from Mann–Whitney U test and considered an absolute value $d\ge 0.3$ as noteworthy. Sub-analyses on each clinical dataset separately are available online in the supplemental materials.

## Results

### REM-OSA distributions

**Fig. 3:**
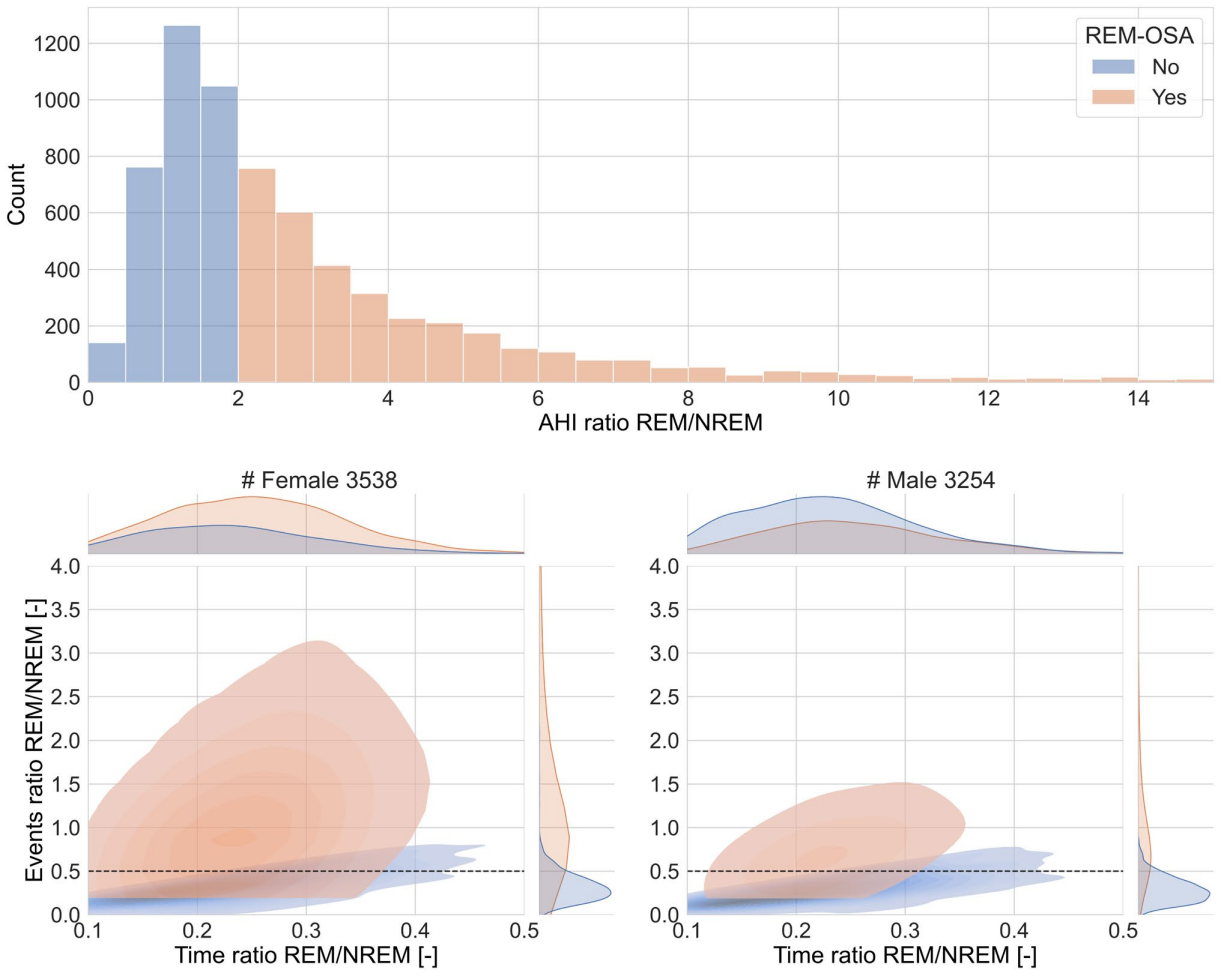
Distribution of AHI ratio in the whole sample grouped by REM-OSA definition. Below, density estimate of *T*_*ratio*_ and *Ev*_*ratio*_ grouped by sex. The dashed line indicates the cutoff for an *Ev*_*ratio*_ of 0.5. The axes are limited to the data in the 5th to 95th percentiles

Figure 3 shows the separation of the analyzed sample using the $AH{I}_{ratio}$ and the general REM-OSA formula, and the same separation in the ${T}_{ratio}$ and $E{v}_{ratio}$ space. The $AH{I}_{ratio}$ separates the two groups by design, as visible in the histogram on the top of the figure. However, when representing the samples in the bidimensional space defined by the time and events ratio, an overlap is visible. Qualitatively, REM-independent-OSA had similar distributions for male and female participants, while REM-OSA showed a wider distribution for females, particularly on the $E{v}_{ratio}$ axis.

**Fig. 4:**
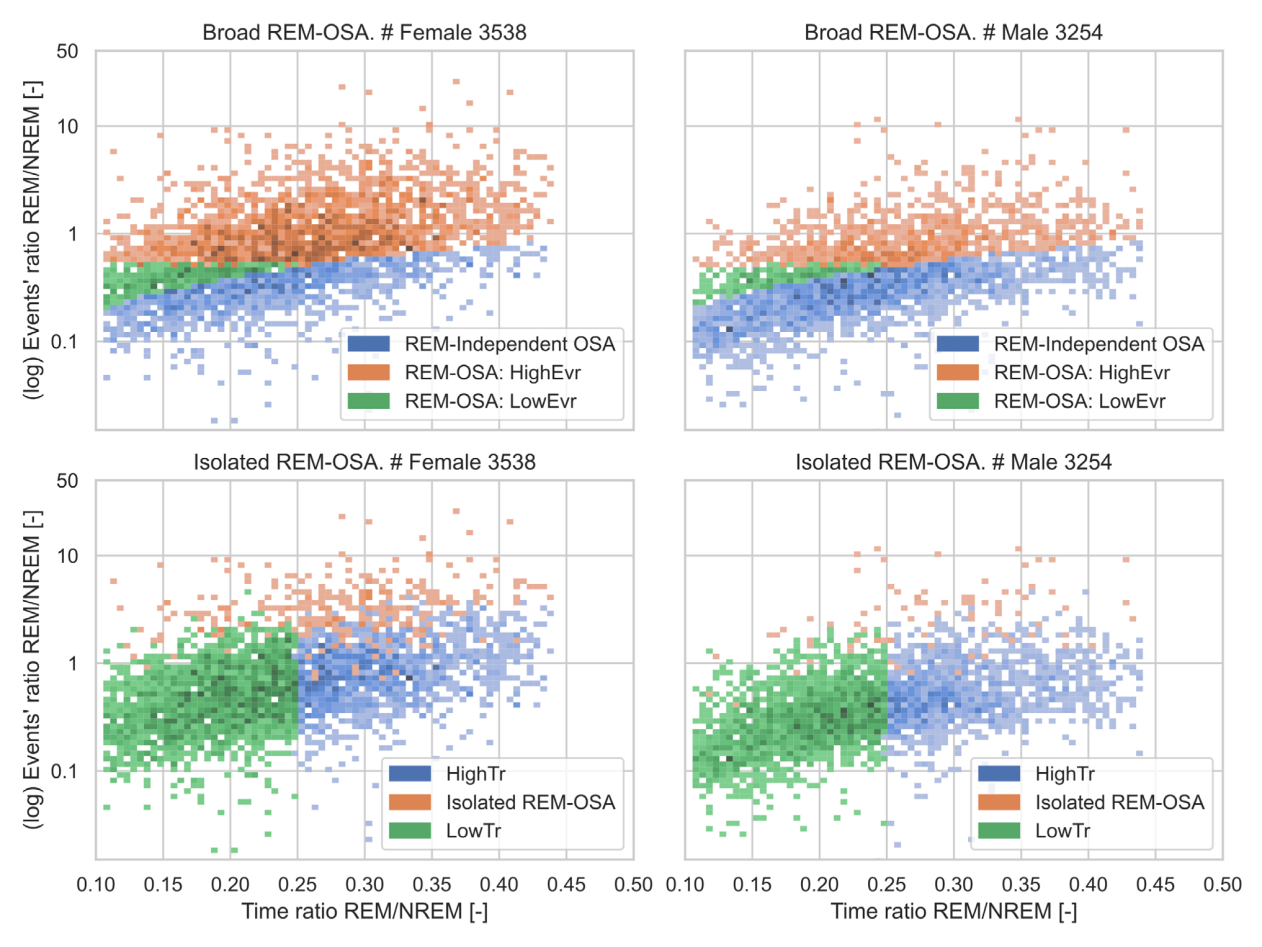
Distribution of time and events (logarithmic) ratios grouped by sex for REM-OSA and *Isolated* REM-OSA. Range limited to 2th and 98th percentile of the whole data

Figure 4 illustrates the whole sample, colored according to the subgroups defined in Sect. 2.4. It is important to observe that the ${T}_{ratio}$ and $E{v}_{ratio}$ space remains relatively uniform, indicating that the subgroups are not based upon trivially separable clusters. However, we can appreciate some qualitative differences.

Using the original REM-OSA definition, participants tend to have either a high $E{v}_{ratio}$, or a low ${T}_{ratio}$, while the simultaneous occurrence of high $E{v}_{ratio}$ and low ${T}_{ratio}$ is much less frequent. Furthermore, we can see that REM-independent-OSA participants rarely have an $E{v}_{ratio}$ higher than 1 (4/3215, 0.12%, only females), hinting a predominance of NREM events. Nevertheless, time spent in REM stage still affects REM-independent-OSA patients, as the $E{v}_{ratio}$ increases from 0.1 with a ${T}_{ratio}$= 0.1 to approximately 0.7 with a ${T}_{ratio}$= 0.4. Using the *isolated* REM-OSA definition, the two REM-independent-OSA subgroups spread over a large space in both the ${T}_{ratio}$ and $E{v}_{ratio}$ axes, but the REM-OSA themselves presented some peculiarities. Despite, by definition, having an $AH{I}_{ratio}\ge 1$ (i.e., $AH{I}_{REM}\ge AH{I}_{NREM}$), a percentage (32/443, 7.2%) of participants classified as *isolated* REM-OSA still had an $E{v}_{ratio}$ lower than 1, i.e., with more events during NREM than during REM.. Additionally, *isolated* REM-OSA participants covered a large ${T}_{ratio}$: 20 (4.5%) spent more than 30% of their sleep in REM, 37 (8.3%) less than 15%.

### REM-OSA analysis

The results of this section are summarized in Table 2. Proportionally, REM-independent-OSA and REM-OSA were almost balanced, with REM-OSA representing 53.4% of the sample. The majority of REM-independent-OSA participants were males (64.2%). In the REM-OSA groups the majority were females, but with a significantly higher proportion in HighEvr(68.2% versus 58.5%).

**Table 2:** Comparison of REM-OSA participants (according to *Haba-Rubio *et al*.* definition [6]) with events’ ratio < 0*.*5 (LowEvr) and high (HighEvr) and REM-independent-OSA participants. Values presented as median (inter-quartile range)

Variable	Group 1 REM-independent	Group 2 LowEvr	Group 3 HighEvr	Effect size (η2)	Cliff's Delta (d+ 1->2)	Cliff's Delta (d+ 2->3)
Demographics						
Sex [F/M]	3217 (1153/2064)	537 (314/223)	3038 (2071/967)	NA	NA	NA
Age [years]	66.00 (15.00)	65.00 (16.00)	61.00 (16.00)	0.026	*0.011 *^*†*^	0.18
BMI [kg/m^2^]	28.04 (5.89)	27.83 (5.90)	26.99 (6.15)	0.012	*0.057 *^*†*^	*0.078 *^*†*^
REM-OSA ratios						
AHI ratio REM/NREM [-]	1.28 (0.65)	2.37 (0.55)	3.92 (3.14)	0.776*	-1.0	-0.716
Resp events ratio REM/NREM (*Ev*_*ratio*_) [-]	0.28 (0.21)	0.40 (0.12)	1.00 (0.89)	0.724*	-0.467	-1.0
TST ratio NREM/REM (*T*_*ratio*_) [-]	0.23 (0.11)	0.16 (0.06)	0.27 (0.10)	0.158*	0.56	-0.819
Sleep features Total Sleep Time (TST) [mins]	380.50 (80.00)	376.50 (79.00)	389.00 (76.00)	0.007	*0.052 *^*†*^	-0.143
Sleep Efficiency [%]	78.62 (14.35)	76.69 (14.23)	80.45 (13.52)	0.011	*0.062 *^*†*^	-0.173
Epworth Sleepiness [ESS]	7.00 (7.00)	7.00 (6.00)	6.00 (6.00)	0.006	0.099	*0.005 *^*†*^
ESS > 10 [#]	819	108	619	NA	NA	NA
Time in stage N1 / TST [%]	18.66 (12.88)	16.37 (10.00)	12.31 (7.61)	0.145*	0.151	0.343
Time in stage N2 / TST [%]	52.28 (12.27)	58.10 (11.16)	53.78 (10.62)	0.031	-0.37	0.289
Time in stage N3 / TST [%]	7.88 (11.31)	9.91 (11.33)	11.46 (10.89)	0.033	-0.124	-0.099
Time in stage REM / TST [%]	18.36 (7.22)	13.84 (4.31)	21.02 (6.15)	0.158*	0.56	-0.819
Time in stage REM [mins]	68.00 (33.00)	50.00 (20.00)	80.50 (31.00)	0.137*	0.494	-0.75
Wake after Sleep Onset (WASO) [mins]	65.00 (62.50)	66.00 (62.50)	52.00 (54.00)	0.023	*-0.016 *^*†*^	0.195
WASO longer than 5 min [mins]	39.00 (56.50)	40.50 (62.00)	29.50 (49.50)	0.012	*-0.025 *^*†*^	0.153
WASO—REM time [mins]	-3.50 (77.00)	14.00 (69.50)	-28.00 (67.50)	0.071*	-0.228	0.488
REM latency [mins]	87.00 (72.00)	100.50 (88.50)	80.00 (50.50)	0.013	-0.133	0.235
Avg. duration of stage REM [mins]	9.38 (5.75)	7.21 (4.09)	10.05 (5.97)	0.035	0.332	-0.425
Awakenings per hour [events/hour]	4.35 (2.44)	4.44 (1.94)	3.67 (1.80)	0.046	*-0.01 *^*†*^	0.282
Arousals per hour [events/hour]	13.44 (9.94)	10.55 (7.38)	9.05 (6.13)	0.119*	0.249	0.19
Position features						
Time in position supine / TST [%]	29.03 (56.08)	26.78 (58.72)	24.51 (55.22)	0.001	*0.004 *^*†*^	*0.043 *^*†*^
Time supine in REM / time in REM [%]	15.24 (54.89)	21.18 (60.95)	18.23 (59.01)	0.000	*-0.035 *^*†*^	*0.023 *^*†*^
OSA features						
AHI [events/hour]	43.53 (28.05)	27.90 (14.34)	21.17 (14.54)	0.317*	0.492	0.339
Percentage hypopnea events [%]	91.47 (17.78)	96.36 (8.01)	97.22 (6.54)	0.109*	-0.305	-0.112
Median duration of resp events Merged [s]	19.00 (6.00)	18.60 (4.80)	18.15 (5.00)	0.014	0.112	*0.036 *^*†*^
SpO2 features						
Desaturations [#]	201.00 (147.00)	136.00 (103.00)	107.00 (83.00)	0.203*	0.334	0.264
Avg. desaturation duration [s]	23.01 (10.05)	22.68 (10.01)	22.15 (7.61)	0.005	*0.026 *^*†*^	0.064
TST under 90% SpO2 (TST90) [mins]	3.47 (18.35)	1.65 (8.25)	0.42 (3.82)	0.08*	0.141	0.223
Area under 90% SpO2 (CA90) [%*mins]	5.31 (38.34)	2.60 (14.82)	0.47 (6.47)	0.074*	0.135	0.214
SpO2 nadir [%]	80.00 (11.33)	79.30 (9.96)	80.08 (10.94)	0.002	*0.01 *^*†*^	-0.069
Hypoxic burden [(%min)/h]	18.15 (30.57)	9.79 (16.37)	6.35 (9.14)	0.175*	0.272	0.271

^+^Cliff’s delta *d* for Mann–Whitney U two populations tests. The header numbers indicate the groups of interest, the sign the direction of the effect (i.e., positive 1 → 2 means group 1 values are more often higher than those in group 2)

*KW: Kruskal–Wallis *p* < *.*001 and effect size *η*^2^ > *.*06

^†^**Not** significant Dunn post-hoc test (*p* > 0*.*01)

^x^BMI not available in MESA dataset

In comparison with the HighEvr subgroup, only excessive sleepiness (ESS) and the duration of respiratory events were not significantly different from LowEvr participants. LowEvr and REM-independent- participants exhibited similarities in some sleep features, such as sleep efficiency, WASO, and the frequency of awakenings. For most characteristics, the LowEvr subgroup showed values between the REM-independent- and the HighEvr subgroups. For example, the AHI in the LowEvr subgroup was substantially higher than the HighEvr one, but lower than REM-independent-OSA. The same phenomenon is noticeable in desaturation features and arousals.

The LowEvr subgroup stood out in terms of REM architecture: the time in REM was the lowest, both in absolute terms and as percentage of TST, and showed the highest latency. The LowEvr subgroup was also the only with a positive average WASO-REM difference, while it was negative in both other subgroups.

We did not observe significant differences in terms of demographic factors (age, BMI), sleep quality (TST, sleep efficiency), time in supine position, SpO2 average and nadir, and average duration of respiratory and desaturation events, both in REM and NREM. Despite similar average ESS, the REM-independent- subgroup had a significantly higher proportion of participants with ESS above 10 (the current clinical threshold for excessive sleepiness). The two REM-OSA subgroups had similar proportions.

### Isolated REM-OSA analysis

The results of this section are summarized in Table 3. The *isolated* REM-OSA subgroup represented less than 7% of the total sample and was composed mostly by females (77.2%). The HighTr group had a slightly, but significantly, higher percentage of females compared to LowTr(52.3% versus 48.9%).

**Table 3:** Comparison of REM-independent-OSA participants (according to *Mokhlesi *et al*.* definition [7]) with REM/NREM time ratio > 0*.*25 (HighTr) and high (LowTr) and *isolated* REM-OSA participants. Values presented as median (inter-quartile range)

Variable	Group 1 Isolated REM-OSA	Group 2 HighTr	Group 3 LowTr	Effect size (η2)	Cliff's Delta (d+ 1->2)	Cliff's Delta (d+ 2->3)
Demographics						
Sex [F/M]	443 (342/101)	2707 (1416/1291)	3642 (1780/1862)	NA	NA	NA
Age [years]	58.00 (15.00)	62.00 (16.00)	65.00 (16.00)	0.026	-0.201	-0.14
BMI [kg/m^2^]	25.61 (5.44)	27.77 (5.96)	27.68 (6.04)	0.013	-0.271	*0.001 *^*†*^
REM-OSA ratios						
AHI ratio REM/NREM [-]	10.58 (7.74)	2.18 (2.08)	1.85 (1.79)	0.167*	0.917	0.128
Resp events ratio REM/NREM (*Ev*_*ratio*_) [-]	2.86 (2.50)	0.69 (0.71)	0.34 (0.36)	0.312*	0.849	0.509
TST ratio NREM/REM (*T*_*ratio*_) [-]	0.28 (0.10)	0.30 (0.07)	0.19 (0.07)	0.703*	-0.308	1.0
Sleep features						
Total Sleep Time (TST) [mins]	393.50 (76.50)	393.00 (75.50)	377.00 (77.00)	0.016	*0.018 *^*†*^	0.147
Sleep Efficiency [%]	80.66 (13.46)	81.30 (12.96)	77.50 (14.22)	0.027	*-0.014 *^*†*^	0.195
Epworth Sleepiness [ESS]	6.00 (6.00)	7.00 (6.00)	7.00 (6.00)	0.001	-0.091	0.042
ESS > 10 [#]	85	640	821	NA	NA	NA
Time in stage N1 / TST [%]	10.99 (6.85)	13.88 (8.92)	17.25 (12.44)	0.065*	-0.265	-0.241
Time in stage N2 / TST [%]	53.95 (9.78)	50.75 (10.09)	55.61 (11.85)	0.065*	0.243	-0.305
Time in stage N3 / TST [%]	11.63 (10.02)	9.69 (11.01)	9.47 (11.98)	0.004	0.161	*0.01 *^*†*^
Time in stage REM / TST [%]	21.65 (6.04)	23.24 (4.28)	15.98 (4.78)	0.703*	-0.308	1.0
Time in stage REM [mins]	83.50 (29.75)	91.50 (22.75)	58.00 (23.00)	0.533*	-0.236	0.868
Wake after Sleep Onset (WASO) [mins]	51.00 (55.75)	51.00 (51.50)	67.00 (62.88)	0.031	*0.003 *^*†*^	-0.206
WASO longer than 5 min [mins]	28.00 (50.00)	28.00 (47.75)	40.50 (58.00)	0.02	*0.013 *^*†*^	-0.169
WASO—REM time [mins]	-32.50 (68.50)	-42.00 (60.00)	8.50 (71.50)	0.211*	0.097	-0.545
REM latency [mins]	83.00 (49.00)	74.50 (46.00)	95.50 (76.50)	0.054	0.145	-0.28
Avg. duration of stage REM [mins]	10.34 (5.59)	11.12 (6.21)	8.22 (4.88)	0.11*	-0.112	0.395
Awakenings per hour [events/hour]	3.48 (1.56)	3.73 (1.88)	4.37 (2.32)	0.046	-0.107	-0.228
Arousals per hour [events/hour]	8.14 (5.60)	10.52 (7.44)	11.70 (9.09)	0.032	-0.296	-0.121
Position features						
Time in position supine / TST [%]	24.25 (51.37)	26.72 (55.70)	27.40 (57.18)	0.000	*-0.046 *^*†*^	*-0.008 *^*†*^
Time supine in REM / time in REM [%]	18.80 (53.52)	15.38 (59.15)	18.46 (55.65)	-0.001	*0.002 *^*†*^	*-0.009 *^*†*^
OSA features						
AHI [events/hour]	9.57 (5.45)	30.21 (21.64)	32.24 (27.42)	0.154*	-0.921	-0.063
Percentage hypopnea events [%]	98.61 (4.63)	95.57 (10.53)	94.31 (13.80)	0.035	0.38	0.084
Median duration of resp events Merged [s]	19.00 (5.58)	18.60 (5.50)	18.90 (5.60)	0.001	*0.056 *^*†*^	-0.051
SpO2 features						
Desaturations [#]	55.00 (35.00)	147.00 (115.00)	161.00 (142.00)	0.107*	-0.746	-0.081
Avg. desaturation duration [s]	22.80 (8.10)	22.09 (8.02)	22.97 (9.84)	0.004	0.08	-0.084
TST under 90% SpO2 (TST90) [mins]	0.03 (0.63)	0.95 (7.87)	2.23 (12.41)	0.046	-0.372	-0.131
Area under 90% SpO2 (CA90) [%*mins]	0.00 (1.01)	1.38 (14.51)	3.18 (25.33)	0.043	-0.37	-0.122
SpO2 nadir [%]	81.25 (12.11)	80.08 (10.94)	80.00 (10.38)	0.002	*0.057 *^*†*^	0.048
Hypoxic burden [(%min)/h]	2.67 (3.88)	9.65 (16.31)	13.21 (26.96)	0.087*	-0.595	-0.148

^+^Cliff’s delta *d* for Mann–Whitney U two populations tests. The header numbers indicate the groups of interest, the sign the direction of the effect (i.e., positive 1 → 2 means group 1 values are more often higher than those in group 2)

^*^KW: Kruskal–Wallis *p* < *.*001 and effect size *η*^2^ > *.*06

^†^**Not** significant Dunn post-hoc test (*p* > 0*.*01)

^x^BMI not available in MESA dataset

Also in this case, the *isolated* REM-OSA and the two REM-independent- subgroups (HighTr and LowTr) were heterogeneous with respect to our features of interest. Differences in REM sleep features are trivial because of the ${T}_{ratio}$ split we employed to separate HighTr and LowTr. Sleep efficiency, AHI and the number of desaturations were substantially similar. However, WASO, frequency of awakenings and desaturation features were substantially worse in LowTr compared to HighTr.

Conversely, HighTr and *isolated* REM-OSA subgroups presented many similarities in terms of sleep architecture (TST, WASO, efficiency, time in REM, etc..) and awakenings. Differences emerged in OSA and desaturation features, where *isolated* REM-OSA participants had a significantly lower overall AHI and hypoxic burden.

We did not observe significant differences in terms of demographic factors (age, BMI), sleep quality (TST, sleep efficiency, ESS), and time in supine position. The proportion of participants with $ESS>10$ in *isolated* REM-OSA was significantly lower than in the HighTr, but not in the LowTr subgroup.

### Effect of biological sex on REM-OSA

Table 4 collates differences between participants of either sex according to both the REM-OSA and *isolated* REM-OSA definitions Overall, we did not observe strong differences in each subgroup. Among the few with a medium to large effect size: female participants spent less time in N1 sleep and more in N3 compared to male participants. Females presented a slightly higher $AH{I}_{ratio}$ and $E{v}_{ratio}$(in line with existing literature), but the effect is most prominent in the HighTr and LowTr subgroups using the *isolated* REM-OSA definition rather than in the sub-groups defined according to the general REM-OSA definition (partially reflected also by the overall AHI effect size). Albeit with a smaller effect size, females presented a lower ESS, less frequent awakenings and arousals, and longer REM bouts (but a higher REM latency).

**Table 4:** Effect of biological sex per group with Cliff’s delta *d* calculated from Mann–Whitney U test (female → male, a positive value indicates stronger effect for females)

Variable	REM-OSA groups			Isolated REM-OSA groups		
	REM-independent	LowEvr	HighEvr	Isolated	HighTr	LowTr
Demographics						
Sex [F/M]	1153/2064	314/223	2071/967	342/101	1416/1291	1780/1862
Age [years]	0.14	0.16	0.05	-0.02	0.02	0.09
BMI [kg/m^2^]	-0.04	0.05	-0.08	-0.16	-0.10	-0.04
REM-OSA ratios						
AHI ratio REM/NREM [-]	0.18	0.22	0.27	0.19	0.40*	0.39*
Resp events ratio REM/NREM (*Ev*_*ratio*_) [-]	0.11	-0.03	0.21	0.16	0.38*	0.35*
TST ratio REM/NREM (*T*_*ratio*_) [-]	0.01	-0.19	-0.04	-0.05	0.03	0.00
Sleep features						
Total Sleep Time (TST) [mins]	0.11	0.18	0.15	0.13	0.17	0.12
Sleep Efficiency [%]	0.01	-0.01	0.03	0.06	0.06	0.01
Epworth Sleepiness [ESS]	-0.13	-0.25	-0.11	-0.14	-0.16	-0.15
ESS > 10 [#]	-0.08	-0.09	-0.08	-0.08	-0.10	-0.08
Time in stage N1 / TST [%]	-0.37*	-0.23	-0.32*	-0.31*	-0.46*	-0.39*
Time in stage N2 / TST [%]	0.08	0.05	-0.02	0.05	0.04	0.10
Time in stage N3 / TST [%]	0.34*	0.27	0.32*	0.20	0.40*	0.34*
Time in stage REM / TST [%]	0.01	-0.19	-0.04	-0.05	0.03	0.00
Time in stage REM [mins]	0.05	-0.07	0.04	0.03	0.14	0.05
Wake after Sleep Onset (WASO) [mins]	-0.06	0.05	-0.05	-0.16	-0.10	-0.06
WASO longer than 5 min [mins]	-0.02	0.05	-0.03	-0.14	-0.07	-0.02
WASO—REM time [mins]	-0.07	0.07	-0.06	-0.11	-0.14	-0.07
REM latency [mins]	0.14	0.20	0.13	0.10	0.13	0.10
Avg. duration of stage REM [mins]	0.21	0.07	0.18	0.15	0.21	0.15
Awakenings per hour [events/hour]	-0.23	-0.08	-0.21	-0.23	-0.26	-0.23
Arousals per hour [events/hour]	-0.12	-0.04	0.02	0.04	-0.13	-0.17
Position features						
Time in position S / TST [%]	0.07	0.13	0.07	-0.04	0.05	0.07
Time supine in REM / time in REM [%]	0.07	0.12	0.07	-0.03	0.07	0.08
OSA features						
AHI [events/hour]	-0.14	0.01	-0.12	0.09	-0.25	-0.29
Percentage hypopnea events [%]	0.16	0.05	0.06	-0.00	0.14	0.23
Median duration of resp events Merged [s]	-0.15	-0.11	-0.11	-0.07	-0.15	-0.18
SpO2 features						
Desaturations [#]	-0.07	0.09	-0.05	0.09	-0.16	-0.18
Avg. desaturation duration [s]	-0.04	0.03	-0.04	-0.03	-0.06	-0.06
TST under 90% SpO2 (TST90) [mins]	-0.11	-0.00	-0.12	-0.11	-0.17	-0.16
Area under 90% SpO2 (CA90) [%*mins]	-0.11	0.00	-0.11	-0.10	-0.16	-0.16
SpO2 nadir [%]	0.01	-0.03	-0.03	-0.02	-0.00	0.00
Hypoxic burden [(%min)/h]	-0.13	0.03	-0.13	-0.06	-0.23	-0.22

^*^Mann-Whitney U *p* < 0*.*001 and absolute Cliff’s delta *d* ≥ 0*.*3, ^*X*^BMI not available in MESA dataset

Overall, we can consider the subgroups generally homogeneous, with biological sex having a limited influence in the differences between each subgroup.

## Discussion

We present a novel analysis of the characteristics of REM-related OSA (REM-OSA). We focused in particular on spurious subgroups in the REM-OSA phenotypes identified by two formulations: the original definition from Haba-Rubio et al*.* which considered only the ratio between AHI during REM and AHI during NREM [6] (here named simply REM-OSA), and the stricter definition by Mokhlesi et al*.* which added requirements regarding a maximum AHI during NREM and a minimum amount of time in REM [7] (*Isolated* REM-OSA). To disentangle the contribution of time and number of events from the criteria, we deconstructed the $AH{I}_{ratio}$ formula in its two components: a REM/NREM time ratio (${T}_{ratio}$) and a REM/NREM event ratio ($E{v}_{ratio}$).

From the resulting equation (Eq. 3, $AH{I}_{ratio}={T}_{ratio}\times E{v}_{ratio}$) we can appreciate two components. The $E{v}_{ratio}$ may be considered OSA-specific, with all the patho-physiological factors influencing the risk of a respiratory event in REM compared to an event in NREM. On the other side, the time ratio ${T}_{ratio}$ may be controlled by other mechanisms of sleep pressure and sleep stage cycling [22, 23] The two components influence each other, with the mere occurrence of REM sleep naturally increasing the risk of respiratory events during REM, and of course, the occurrence of respiratory events in specific sleep stages altering what would otherwise be a normal sleep architecture.

With this new interpretation, we can appreciate that a person may be classified with REM-OSA in two different ways: 1) an actual disproportionate amount of respiratory events during REM, 2) less events in REM than in NREM, but with an $AH{I}_{REM}$ amplified by a disproportionately low time in REM sleep.

Considering the broader REM-OSA definition, the second category, here named *LowEvr*, not only exhibited significant differences with respect to the *HighEvr* subgroup, but also some similarities with the REM-independent-OSA group. The new LowEvr subgroup is characterized by an evident reduction in the percentage of REM time ($<15\hspace{0.25em}\mathrm{\%}$) compared to REM-independent- and HighEvr subgroups, presenting higher REM latency and shorter REM bouts. We also observed that the difference between WASO and REM time was positive in this LowEvr subgroup, and negative on the others, suggesting that WASO might have disproportionately impacted REM sleep in these patients more than in the others. Although we did not explore the causes of this change in REM architecture, we hypothesize that this group presents higher arousability from REM sleep, or that awakenings in REM lead to longer bouts awake compared to NREM. The variability of our results remain large (around 70 min), therefore in each subgroup there may be patients were WASO is preceded more by NREM than REM, due to mechanisms independent from REM-OSA. A more in-depth detailed analyses of the sleep architecture, or of the effects of concomitant insomnia diagnoses (also known as COMISA), of these patients is necessary to confirm these hypotheses. At the same time, the LowEvr subgroup presents more desaturations and a higher hypoxic burden compared to HighEvr. Both REM sleep loss and higher hypoxic burden are known to increase cardiovascular risk [18, 24]. Therefore, the combination of these phenomena may explain the higher cardiovascular risk seen in REM-OSA [3]. Future studies with longitudinal datasets (like the SHHS and MESA cohorts employed in the present study) could examine whether this new phenotype is more vulnerable than the REM-OSA subgroup with preserved REM time.

The low specificity of the original REM-OSA formulation has been already dissected in other research works, leading to stricter definitions of REM-OSA by Conwell et al*.* [9] and by Mokhlesi et al*.* (*Isolated* REM-OSA [7]) We analyzed *isolated* REM-OSA groups to investigate if the decomposition along the ${T}_{ratio}$ and $E{v}_{ratio}$ provided new insights. The first observation is that some participants classified as *isolated* REM-OSA have a large spread in ${T}_{ratio}$ and in some cases had an $E{v}_{ratio}<1$, meaning that they had more events during NREM than during REM sleep. Consequently, their classification as REM-OSA may have been caused by a pathological sleep architecture (low REM/excessive NREM) rather than a higher risk of respiratory events during REM. Second, the REM-independent-OSA(non-*isolated* REM-OSA) subgroup overlapped all subgroups defined according to the general REM-OSA criteria. This overlap implies that we could extract two highly heterogeneous "REM-independent-OSA" subgroups by setting an arbitrary threshold over the REM/NREM time ratio. These subgroups (which we named *HighTr* and *LowTr*) presented similarities and differences with the *isolated* REM-OSA group and between each other. For example, the REM and wakefulness architecture in HighTr is closer to that of the *isolated* REM-OSA subgroup, but at the same time, the number of desaturations and hypoxic burden are closer to those in LowTr.

Our analyses also show that certain subgroups, namely *isolated* REM-OSA and HighEvr in the REM-OSA criteria, are associated with the female population and present a lower overall AHI. At the same time, differences between males and females in those subgroups seem limited, and other subgroups with higher AHI share certain characteristics. Future research should consider both the overall risk of respiratory events, potential alterations in sleep architecture, and arousability to truly understand the effect of biological sex on REM-OSA.

Lastly, we must consider that there is an interplay between REM sleep and supine position in increasing the risk of respiratory events. Despite non significant differences between our groups of interest, the overall distribution of time in supine position (both over the whole night and in REM) covers a range from 0 to around 70%. Therefore, we can hypothesize that time in supine position may partially explain the variability of AHI in all our groups. Vice-versa, other OSA patients may spend more time in lateral positions, but present severe cardiovascular co-morbidities that are only loosely dependent from their OSA severity [25]. Future research will explore the combined effect of sleep stages and body position together. For example, the development of simulation models, such as those in *Eiseman *et al*.* [12], may allow a better estimation of the risk (or severity) of events dependent from supine position and REM sleep.

Although the usage of three large datasets makes our results more general, this work is not exempt from limitations. The relatively older age of the participants in the datasets does not allow us to estimate the prevalence of REM-reduced phenotypes in a younger population. Aging alone can lead to a reduction of overall REM sleep [26], so future work should investigate whether our findings hold in younger (or even pediatric) OSA populations. Additionally, it is worthwhile remembering that OSA is a disease often accompanied by a variety of co-morbidities that may be treated with prescription drugs that may alter sleep architecture and change the risk of sleep apnea. Although we excluded participants with other diagnosed sleep disorders, our current analysis did not control for REM-altering or apnea-inducing drugs (including also smoking and alcohol consumption). Similarly, older OSA patients and smokers may be more prone to COPD overlap syndrome, which may affect sleep architecture, $AH{I}_{REM}$ and time below 90% oxygen saturation [27] The datasets had some differences regarding how the data was collected, sample size, demographics, sleep and OSA metrics. Despite these differences, nearly all subgroups’ characteristics we encountered in the aggregated dataset were also present (as quantified by statistical tests and their effect sizes) in each individual dataset, as detailed in supplemental materials. This support the generalizability of our findings across different cohorts. Further studies with a higher control of confounders and causal risk factors of REM-OSA are hence necessary.

It is important to emphasize that we do not advocate the direct usage of our set of (arbitrary) thresholds (see Appendix 1.6 for assumptions on clustering thresholds) or new formulations based on ${T}_{ratio}$ and $E{v}_{ratio}$ space to define REM-OSA patients. Instead, we hope to provide a new tool to explore and understand how the probability of REM events is intrinsically conditioned by the relative time spent in REM. Our work follows the trail of others, recognizing the limitations of AHI-based metrics [28]. For example, previous literature expanded on the idea of AHI from a fixed constant rate to a distribution, enabling a more robust evaluation of REM dominance [29]. Other authors evolved that into an apnea intensity model that takes in consideration sleep stages, body position, and recurrence of respiratory events [30]. Other methods may consider risk ratios as a simple modeling mechanism, with REM sleep being the exposure factor and the count of events as the outcome. Here, we presented how the $AH{I}_{ratio}$, employed so far to characterize REM-OSA, is by definition a two-dimensional space which could be analyzed differently.

Specifically, a two-dimensional space could be used to expand the dichotomy of REM-OSA and REM-independent-OSA in what could be a differentiator between REM-prevalent OSA, NREM-prevalent OSA and a truly REM-independent, or better named *sleep stage-neutral*, OSA. The correct identification of a REM-OSA phenotype, or the identification of truly REM-independent-OSA patients may open avenues to research different therapeutic options, or lead to a better understanding of the pathophysiology of the condition, and health consequences. For example, combining OSA therapy (CPAP or other devices) with pharmacological therapies may help reduce apneic events, but also restore a healthy REM architecture. A better interpretation of REM-OSA, including reduced REM time, the presence of hypoxic and cardiovascular markers, could also help us understand why REM-OSA is associated to severe cardio-metabolic consequences, despite an overall lower AHI.

## Conclusions

In this paper, we present a novel representation of REM-OSA criteria in bidimensional space defined by a time and events ratio. This representation allowed us to identify potential phenotypes with marked pathophysiological differences. Despite some limitations, we advocate for a critical appraisal of methodologies that are based only on AHI ratio thresholds, and we hope that our representation could ignite new approaches and useful clinical results in the complex field of REM-OSA research, possibly leading to clinically useful definitions of new REM-OSA phenotypes.

## Electronic supplementary material

Below is the link to the electronic supplementary material.

Supplementary file1 (PDF 168 KB)

## Figures and Tables

**Fig. 5:**
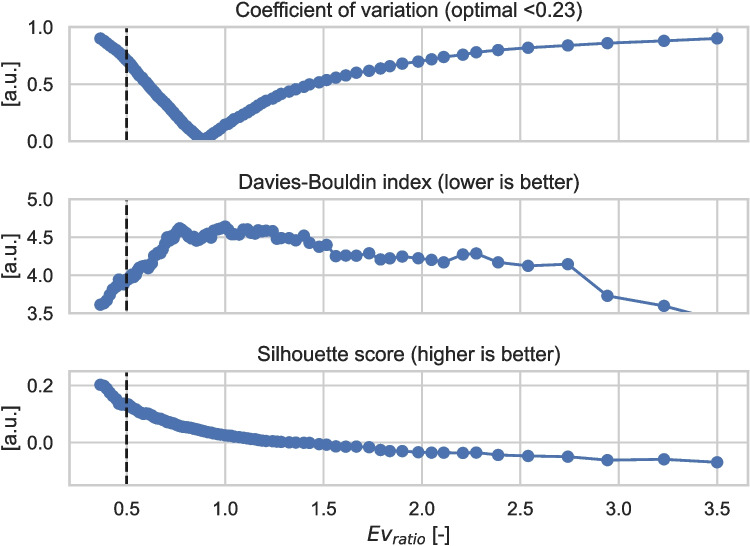
Clustering evaluation of REM-OSA subgroups with changing Evratio. As we mentioned in the Sect. 4 of the main text, it is unlikely that thresholds based on a pair of Evratio and Tratio would provide perfect clusters. More likely, the ratios’ space provides a better starting point for clustering methods. Different clustering techniques may divide this space along linear thresholds or arbitrary clusters. Future research will explore automatic clustering techniques based upon Evratio and Tratio space

**Fig. 6:**
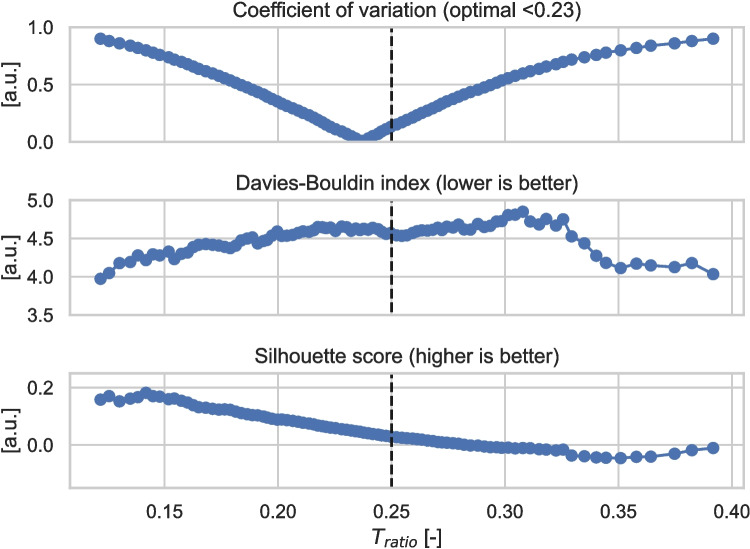
Clustering evaluation of Isolated REM-OSA REM-independent subgroups with changingTratio.

## Data Availability

The SOMNIA data used in this study are available from the Sleep Medicine Centre Kempenhaeghe upon reasonable request. The data can be requested by presenting a scientific research question and by fulfilling all the regulations concerning the sharing of the human data. The details of the agreement will depend on the purpose of the data request and the entity that is requesting the data (e.g. research institute or corporate). Each request will be evaluated by the Kempenhaeghe Research Board and, depending on the request, approval from independent medical ethical committee might be required. Access to data from outside the European Union will further depend on the expected duration of the activity; due to the work required from a regulatory point of view, the data is less suitable for activities that are time critical, or require access in short notice. Specific restrictions apply to the availability of the data collected with sensors not comprised in the standard PSG set-up, since these sensors are used under license and are not publicly available. These data may however be available from the authors with permission of the licensors. For inquiries regarding availability, please contact Merel van Gilst (M.M.v.Gilst@tue.nl). SHHS and MESA datasets are available upon request at NSRR (https://www.sleepdata.org/).

## Appendix

### A evaluation of Ev_ratio_ and T_ratio_ thresholds

In our analysis, we selected two thresholds for *Evratio* and *Tratio* to separate the REMOSA and isolated REM-independent-OSA subgroups, respectively. The selection of an *Evratio* = 0.5 and *Tratio* = 4 (as described in Sect. 2.4 in the main text) were arbitrary and determined mostly by the average time spent in REM sleep as percentage of total sleep time (TST) around 20%. This value leads to a *Tratio* of 0.25, and substituting it in Eq. 3, it leads to an *Evratio* = 0.5 so that *AHIratio* ≥ 2. However, as we observed in the Sect. 3.1, the whole sample is relatively homogeneous in the ratios’ space, so that a pair of thresholds that perfectly divide the data in good clusters may not exist. In this appendix we evaluate how different *Evratio* and *Tratio* affect our subgroups of interest. To do so, we separated the subgroups with a moving threshold from the 5th to the 95th percentile of *Evratio* and *Tratio*. In our dataset these ranges were 0.36-3.5 and 0.12–0.39 for *Evratio* and *Tratio*, respectively. In the REM-OSA analysis, we Have separated the REM-OSA participants, while in the isolated REM-OSA analysis, we separated the REM-independent-OSA participants. Then we evaluated the resulting clusters with three common quality metrics:

- Coefficient of variation (known also as cluster size variation).
- Davies-Bouldin index.
- Silhouette score.

The coefficient of variation is the ratio between the sample (in our case, the clusters size) standard deviation and the sample average. Eldrige et al. [1] employed it to evaluate the presence of uneven cluster size, that may affect statistical analyses and effect size calculations. In that paper, a coefficient below 0.23 is considered preferable. In our case, it is not strictly necessary to minimize the coefficient of variation, as we. are interested in the clinical specificity of subgroups. The Davies-Bouldin index [2] measures the average similarity between clusters. A lower index (minimum 0, maximum unbounded) indicate a higher dissimilarity, and potentially a better clustering structure. The silhouette measures the similarity, or cohesion, of a cluster compared to the others. The average silhouette of all clusters (score) is bound between -1 and 1, with higher positive values indicating a better clustering. Both the Davies-Bouldin index and the silhouette score are calculated using the same variables we employed in the Sect. 3 for our statistical comparisons. The evaluation of REM-OSA subgroups is available in Fig. 5. The threshold set at 0.5 has a good balance in both the Davies-Bouldin index and Silhouette score. The coefficient of variation is relatively large, but a higher threshold would worsen the other two factors. If we consider the”REM-independent-OSA” sample that we derived from the isolated REM-OSA criteria and move *Tratio* we obtain the scores available in Fig. 6. In these clusters, the Davies-Bouldin index remains relatively constant in the range 0.18–0.28 of *Tratio*. The two clusters would have a similar size in the that range, as we have seen in Sect. 3.3 in the main text and confirmed by the coefficient of variation here. The silhouette score decreases with *Trati*o. Potentially, a threshold between 0.18 and 0.22 could be a better choice.
