# Supplementary material for: Breathtaking dreams: reduced REM phenotype in REM-related sleep apnea
Source: Sleep Breath. 2025 Jan 22;29(1):87. doi: 10.1007/s11325-024-03236-5 (PMC11754315; doi:10.1007/s11325-024-03236-5)
Supplement: Supplementary file 1 — Supplementary file1 (PDF 168 KB) [file 11325_2024_3236_MOESM1_ESM.pdf]

S1: Dataset comparisons

This document contains the supplemental materials for the paper "Breathtaking dreams: Reduced REM phenotype in REM-related Sleep Apnea" by Cerina et al., 2024

All values in the tables are represented as median (standard deviation) unless stated otherwise.

Symbols in tables are:

- +: Cliff's delta d for Mann-Whitney U two populations tests. The header numbers indicate the groups of interest, the sign the direction of the effect (i.e., positive $1 \rightarrow 2$ means group 1 values are more often higher than those in group 2)
- * **KW**: Kruskal-Wallis p < .001 and effect size $\eta^2 > .06$
- † **Not** significant Dunn post-hoc test (p > 0.01) Numerical differences exist between the three analyzed datasets, but significance levels are overall consistent with the pooled data presented in the main text.
- * (In Cliff's Delta tables) Mann-Whitney U $p < 0.001$ and absolute Cliff's delta $d \geq 0.3$

For each dataset there are three tables, following the structure of results in the main manuscript:

- Comparison of REM-OSA participants (according to *Haba-Rubio et al.*, 2005 definition) with events' ratio < 0.5 (LowEvr) and high (HighEvr) and REM-independent-OSA participants. Values presented as median (interquartile range)
- Comparison of REM-independent-OSA participants (according to *Mokhlesi et al.*, 2012 definition) with REM/NREM time ratio > 0.25 (HighTr) and high (LowTr) and *isolated* REM-OSA participants. Values presented as median (interquartile range).
- Effect of biological sex per group with Cliff's delta d calculated from Mann-Whitney U test (female $\rightarrow$ male, a positive value indicates stronger effect for females).

S1.1 dataset SHHS

Variable	Group 1 REM-independent	Group 2 LowEvr	Group 3 HighEvr	Effect size (η^2, Cliff's delta) η^2 KW	d$^+$ 1→2	d$^+$ 2→3
**Demographics**						
Sex [F/M]	2374 (838/1536)	385 (215/170)	2467 (1688/779)	NA	NA	NA
Age [years]	65.00 (16.00)	64.00 (16.00)	60.00 (16.00)	0.035	*0.028*†	0.191
BMI [kg/m^2]	28.09 (5.96)	27.70 (5.89)	26.96 (6.08)	0.014	*0.076*†	*0.068*†
AHI [events/hour]	44.38 (27.61)	28.24 (14.45)	21.51 (14.42)	**0.343***	0.53	0.328
**REM-OSA ratios**						
AHI ratio REM/NREM [-]	1.31 (0.63)	2.35 (0.54)	3.92 (3.02)	**0.773***	-1.0	-0.724
Resp events ratio REM/NREM (Ev_{ratio}) [-]	0.29 (0.20)	0.40 (0.13)	1.02 (0.89)	**0.724***	-0.425	-1.0
TST ratio NREM/REM (T_{ratio}) [-]	0.23 (0.11)	0.16 (0.06)	0.27 (0.10)	**0.155***	0.582	-0.825
**Sleep features**						
Total Sleep Time (TST) [mins]	379.50 (77.88)	373.00 (75.00)	389.00 (74.00)	0.01	0.088	-0.191
Sleep Efficiency [%]	79.83 (13.07)	78.28 (13.30)	81.77 (12.67)	0.011	*0.043*†	-0.161
Epworth Sleepiness [ESS]	8.00 (6.00)	7.00 (5.00)	7.00 (6.00)	0.01	0.081	*0.043*†
ESS > 10 [#]	662	86	521	NA	NA	NA
Time in stage N1 / TST [%]	18.45 (12.28)	16.13 (10.18)	12.23 (7.38)	**0.146***	0.152	0.332
Time in stage N2 / TST [%]	52.75 (12.24)	59.53 (10.32)	54.07 (10.39)	0.032	-0.399	0.345
Time in stage N3 / TST [%]	7.34 (10.99)	9.55 (10.71)	11.22 (10.77)	0.04	-0.109	-0.141
Time in stage REM / TST [%]	18.72 (7.22)	13.92 (4.40)	21.24 (6.14)	**0.155***	0.582	-0.825
Time in stage REM [mins]	69.00 (32.50)	50.00 (20.50)	81.50 (30.50)	**0.139***	0.527	-0.768
Wake after Sleep Onset (WASO) [mins]	60.50 (57.50)	61.00 (57.00)	49.00 (51.00)	0.02	*-0.012*†	0.181
WASO longer than 5 minutes [mins]	36.00 (53.00)	36.50 (55.50)	28.00 (47.00)	0.011	*-0.018*†	0.14
WASO - REM time [mins]	-9.50 (73.00)	9.50 (59.50)	-32.00 (64.75)	**0.072***	-0.251	0.504
REM latency [mins]	84.50 (66.38)	94.50 (87.50)	79.00 (48.75)	0.009	-0.134	0.211
Avg. duration of stage REM [mins]	9.56 (5.80)	7.50 (4.14)	10.20 (5.70)	0.034	0.34	-0.431
Awakenings per hour [events/hour]	4.10 (2.19)	4.30 (1.79)	3.56 (1.69)	0.039	*-0.046*†	0.289
Arousals per hour [events/hour]	13.17 (9.41)	10.60 (7.04)	9.27 (5.94)	**0.107***	0.234	0.185
**Position features**						
Time in position S / TST [%]	25.44 (58.81)	24.44 (60.55)	22.68 (55.67)	-0.0	*0.017*†	*0.01*†
Time supine in REM / time in REM [%]	13.04 (55.66)	8.98 (57.02)	17.04 (58.19)	-0.001	*0.005*†	*-0.015*†
**OSA features**						
Percentage hypopnea events [%]	90.76 (18.83)	96.27 (7.71)	97.14 (6.90)	**0.121***	-0.33	-0.114
Median duration of resp events Merged [s]	19.00 (6.00)	18.75 (5.10)	18.30 (5.10)	0.007	*0.069*†	*0.039*†
**SpO2 features**						
Desaturations [#]	184.00 (110.00)	119.00 (69.00)	99.00 (68.00)	**0.287***	0.504	0.242
Avg. desaturation duration [s]	20.96 (6.20)	20.66 (5.23)	20.95 (5.62)	-0.0	*0.045*†	*-0.026*†
TST under 90% SpO2 (TST90) [mins]	2.72 (14.95)	1.12 (5.80)	0.30 (2.59)	**0.082***	0.172	0.192
Area under 90% SpO2 (CA90) [%*mins]	4.70 (35.17)	1.83 (13.62)	0.39 (5.45)	**0.077***	0.164	0.187
SpO2 nadir [%]	79.30 (11.33)	79.30 (8.98)	79.30 (10.16)	0.001	*0.001*†	*0.048*†
Hypoxic burden [(%min)/h]	13.63 (16.30)	7.25 (6.94)	5.25 (5.71)	**0.251***	0.435	0.255

Variable	Group 1<br>Isolated REM-OSA	Group 2<br>HighTr	Group 3<br>LowTr	Effect size (η^2, Cliff's delta)<br>η^2 KW	 d^+ 1→2	 d^+ 2→3
**Demographics**						
Sex [F/M]	339 (269/70)	2207 (1168/1039)	2680 (1304/1376)	NA	NA	NA
Age [years]	56.00 (15.00)	61.00 (16.00)	64.00 (17.00)	0.027	-0.229	-0.132
BMI [kg/m^2]	25.50 (5.42)	27.80 (5.96)	27.65 (6.08)	0.015	-0.302	*0.007*†
AHI [events/hour]	10.12 (5.25)	30.33 (21.92)	32.39 (27.19)	**0.154***	-0.919	-0.063
**REM-OSA ratios**						
AHI ratio REM/NREM [-]	10.71 (7.11)	2.26 (2.16)	1.89 (1.87)	**0.17***	0.927	0.135
Resp events ratio REM/NREM (Ev_{ratio}) [-]	3.04 (2.48)	0.71 (0.72)	0.35 (0.38)	**0.319***	0.861	0.515
TST ratio NREM/REM (T_{ratio}) [-]	0.29 (0.10)	0.31 (0.07)	0.19 (0.07)	**0.71***	-0.268	1.0
**Sleep features**						
Total Sleep Time (TST) [mins]	389.50 (75.50)	393.00 (73.00)	375.00 (74.00)	0.021	*-0.017*†	0.172
Sleep Efficiency [%]	82.15 (12.87)	82.36 (12.10)	79.01 (13.28)	0.027	*-0.019*†	0.195
Epworth Sleepiness [ESS]	6.00 (6.00)	7.00 (6.00)	7.00 (6.00)	0.001	-0.113	*0.013*†
ESS > 10 [#]	65	546	658	NA	NA	NA
Time in stage N1 / TST [%]	10.93 (6.65)	13.45 (8.75)	17.02 (11.85)	**0.066***	-0.256	-0.246
Time in stage N2 / TST [%]	54.04 (9.92)	51.22 (9.93)	56.37 (11.71)	**0.074***	0.21	-0.325
Time in stage N3 / TST [%]	11.25 (10.12)	9.44 (10.86)	9.06 (11.59)	0.005	0.157	*0.026*†
Time in stage REM / TST [%]	22.15 (6.08)	23.35 (4.31)	16.13 (4.62)	**0.71***	-0.269	1.0
Time in stage REM [mins]	85.00 (29.25)	92.00 (22.50)	58.00 (22.00)	**0.549***	-0.233	0.877
Wake after Sleep Onset (WASO) [mins]	45.50 (46.75)	48.50 (49.00)	63.00 (59.50)	0.031	*-0.024*†	-0.202
WASO longer than 5 minutes [mins]	26.00 (44.50)	26.50 (44.50)	38.00 (54.50)	0.022	*-0.005*†	-0.172
WASO - REM time [mins]	-37.00 (61.75)	-45.00 (57.50)	4.00 (66.00)	**0.227***	0.089	-0.561
REM latency [mins]	81.50 (47.25)	72.50 (43.00)	93.00 (71.62)	0.055	0.152	-0.282
Avg. duration of stage REM [mins]	10.33 (5.50)	11.24 (6.05)	8.43 (4.92)	**0.106***	-0.119	0.386
Awakenings per hour [events/hour]	3.31 (1.54)	3.61 (1.75)	4.14 (2.11)	0.04	-0.1	-0.216
Arousals per hour [events/hour]	8.14 (5.33)	10.41 (7.06)	11.69 (8.78)	0.034	-0.295	-0.133
**Position features**						
Time in position S / TST [%]	17.86 (51.39)	24.83 (56.99)	24.07 (58.44)	0.0	*-0.071*†	*0.012*†
Time supine in REM / time in REM [%]	14.98 (50.42)	17.43 (55.71)	12.11 (58.81)	-0.0	*-0.032*†	*0.025*†
**OSA features**						
Percentage hypopnea events [%]	98.67 (3.85)	95.48 (10.75)	94.08 (14.52)	0.043	0.422	0.095
Median duration of resp events Merged [s]	19.30 (5.10)	18.40 (5.40)	18.90 (5.60)	0.003	0.11	-0.06
**SpO2 features**						
Desaturations [#]	51.00 (28.00)	137.00 (94.00)	140.00 (106.00)	**0.138***	-0.878	*-0.037*†
Avg. desaturation duration [s]	21.88 (5.60)	20.85 (5.91)	20.90 (5.85)	0.0	*0.08*†	*-0.017*†
TST under 90% SpO2 (TST90) [mins]	0.00 (0.32)	0.68 (5.78)	1.55 (10.02)	0.047	-0.402	-0.122
Area under 90% SpO2 (CA90) [%*mins]	0.00 (0.57)	1.11 (12.13)	2.66 (22.40)	0.045	-0.389	-0.12
SpO2 nadir [%]	80.08 (12.11)	80.08 (10.16)	79.30 (10.16)	0.001	*0.006*†	0.052
Hypoxic burden [(%min)/h]	2.12 (1.90)	7.82 (9.27)	9.38 (12.51)	**0.123***	-0.785	-0.106

Variable	REM-OSA groups			Isolated REM-OSA groups		
	REM-independent	LowEvr	HighEvr	Isolated	HighTr	LowTr
**Demographics**						
Sex [F/M]	838/1536	215/170	1688/779	269/70	1168/1039	1304/1376
Age [years]	0.12	0.11	0.05	-0.11	-0.00	0.08
AHI [events/hour]	-0.18	-0.07	-0.15	0.08	-0.28	**-0.34***
BMI [kg/m^2]	-0.04	0.04	-0.08	-0.15	-0.10	-0.04
**REM-OSA ratios**						
AHI ratio REM/NREM [-]	0.15	0.26	0.27	0.21	**0.39***	**0.39***
Resp events ratio REM/NREM (Ev_{ratio}) [-]	0.09	-0.05	0.22	0.18	**0.38***	**0.34***
TST ratio NREM/REM (T_{ratio}) [-]	0.01	-0.23	-0.03	-0.02	0.03	-0.01
**Sleep features**						
Total Sleep Time (TST) [mins]	0.11	0.16	0.13	0.14	0.17	0.10
Sleep Efficiency [%]	0.05	-0.01	0.04	0.10	0.09	0.03
Epworth Sleepiness [ESS]	-0.12	-0.16	-0.13	-0.19	-0.16	-0.14
ESS > 10 [#]	-0.07	-0.05	-0.10	-0.15	-0.10	-0.08
Time in stage N1 / TST [%]	**-0.41***	-0.23	**-0.31***	**-0.33***	**-0.46***	**-0.41***
Time in stage N2 / TST [%]	0.09	0.13	-0.02	0.02	0.04	0.11
Time in stage N3 / TST [%]	**0.36***	0.22	**0.32***	0.22	**0.40***	**0.35***
Time in stage REM / TST [%]	0.01	-0.23	-0.03	-0.02	0.03	-0.01
Time in stage REM [mins]	0.04	-0.11	0.03	0.04	0.14	0.03
Wake after Sleep Onset (WASO) [mins]	-0.07	0.00	-0.05	-0.18	-0.11	-0.06
WASO longer than 5 minutes [mins]	-0.03	0.00	-0.03	-0.17	-0.08	-0.01
WASO - REM time [mins]	-0.08	0.05	-0.06	-0.15	-0.15	-0.06
REM latency [mins]	0.10	0.17	0.11	0.09	0.11	0.09
Avg. duration of stage REM [mins]	0.20	0.06	0.19	0.13	0.20	0.16
Awakenings per hour [events/hour]	-0.25	-0.09	-0.21	-0.22	-0.25	-0.25
Arousals per hour [events/hour]	-0.10	-0.02	0.04	0.05	-0.10	-0.15
**Position features**						
Time in position S / TST [%]	0.08	0.12	0.04	-0.05	0.04	0.08
Time supine in REM / time in REM [%]	0.07	0.15	0.03	-0.07	0.05	0.08
**OSA features**						
Percentage hypopnea events [%]	0.14	-0.02	0.09	0.05	0.15	0.22
Median duration of resp events Merged [s]	-0.12	-0.08	-0.08	-0.01	-0.13	-0.14
**SpO2 features**						
Desaturations [#]	-0.16	-0.05	-0.09	0.13	-0.23	**-0.30***
Avg. desaturation duration [s]	-0.07	-0.07	-0.02	0.11	-0.06	-0.06
TST under 90% SpO2 (TST90) [mins]	-0.15	-0.06	-0.15	-0.09	-0.20	-0.21
Area under 90% SpO2 (CA90) [%*mins]	-0.14	-0.05	-0.14	-0.09	-0.19	-0.20
SpO2 nadir [%]	0.04	0.02	-0.02	-0.01	0.01	0.04
Hypoxic burden [(%min)/h]	-0.22	-0.11	-0.18	0.04	-0.30	**-0.33***

S1.2 dataset MESA

Variable	Group 1<br>REM-independent	Group 2<br>LowEvr	Group 3<br>HighEvr	Effect size (η^2, Cliff's delta)<br>η^2 KW	 d^+ 1→2	 d^+ 2→3
**Demographics**						
Sex [F/M]	637 (270/367)	130 (91/39)	502 (348/154)	NA	NA	NA
Age [years]	70.00 (14.00)	68.00 (14.00)	66.00 (14.00)	0.015	*0.067*†	*0.102*†
AHI [events/hour]	45.65 (26.74)	27.70 (13.83)	20.88 (14.55)	**0.353***	0.528	0.381
**REM-OSA ratios**						
AHI ratio REM/NREM [-]	1.29 (0.66)	2.47 (0.62)	3.91 (3.23)	**0.777***	-1.0	-0.694
Resp events ratio REM/NREM (Ev_{ratio}) [-]	0.26 (0.19)	0.40 (0.11)	0.94 (0.82)	**0.713***	-0.564	-1.0
TST ratio NREM/REM (T_{ratio}) [-]	0.21 (0.11)	0.16 (0.05)	0.25 (0.10)	**0.148***	0.446	-0.76
**Sleep features**						
Total Sleep Time (TST) [mins]	374.00 (87.00)	386.50 (77.50)	387.25 (85.00)	0.005	*-0.108*†	*-0.007*†
Sleep Efficiency [%]	71.23 (14.74)	71.36 (12.71)	73.50 (13.44)	-0.0	*-0.022*†	*-0.052*†
Epworth Sleepiness [ESS]	5.00 (5.00)	5.00 (4.00)	5.00 (5.00)	0.001	*0.123*†	*0.088*†
ESS > 10 [#]	88	12	75	NA	NA	NA
Time in stage N1 / TST [%]	20.91 (14.92)	17.59 (9.45)	13.35 (9.69)	**0.152***	0.238	0.304
Time in stage N2 / TST [%]	50.82 (12.27)	56.42 (11.28)	52.04 (11.62)	0.038	-0.357	0.208
Time in stage N3 / TST [%]	8.75 (11.91)	11.43 (12.52)	12.80 (11.29)	0.034	-0.19	*-0.035*†
Time in stage REM / TST [%]	17.17 (7.56)	13.77 (3.86)	19.85 (6.48)	**0.148***	0.447	-0.76
Time in stage REM [mins]	62.50 (33.00)	52.00 (17.88)	75.25 (31.00)	**0.119***	0.317	-0.645
Wake after Sleep Onset (WASO) [mins]	85.50 (74.50)	84.50 (76.50)	65.50 (66.00)	0.02	*0.027*†	0.153
WASO longer than 5 minutes [mins]	51.50 (72.00)	56.25 (77.12)	35.00 (63.88)	0.01	*-0.005*†	*0.124*†
WASO - REM time [mins]	20.00 (84.50)	33.50 (83.88)	-7.00 (76.00)	0.054	*-0.104*†	0.373
REM latency [mins]	100.50 (90.50)	113.00 (90.00)	83.00 (66.25)	0.015	*-0.078*†	0.22
Avg. duration of stage REM [mins]	8.21 (5.61)	6.50 (4.15)	9.00 (6.26)	0.025	0.233	-0.337
Awakenings per hour [events/hour]	5.42 (3.27)	4.87 (1.98)	4.47 (2.11)	0.06	0.183	*0.158*†
Arousals per hour [events/hour]	13.04 (10.77)	9.50 (6.04)	7.73 (6.08)	**0.147***	0.318	0.197
**Position features**						
Time in position S / TST [%]	36.21 (49.77)	35.19 (51.24)	31.26 (47.81)	0.004	*0.01*†	*0.089*†
Time supine in REM / time in REM [%]	19.64 (56.80)	30.14 (70.56)	21.48 (61.86)	0.0	*-0.115*†	*0.099*†
**OSA features**						
Percentage hypopnea events [%]	93.33 (13.32)	96.80 (7.13)	97.61 (5.77)	**0.089***	-0.273	*-0.102*†
Median duration of resp events Merged [s]	19.35 (6.00)	17.93 (4.57)	17.50 (4.48)	0.054	0.227	*0.074*†
**SpO2 features**						
Desaturations [#]	369.00 (226.00)	262.00 (158.25)	221.00 (165.75)	**0.189***	0.345	0.248
Avg. desaturation duration [s]	34.58 (8.68)	33.23 (6.93)	32.71 (7.93)	0.006	*0.1*†	*0.017*†
TST under 90% SpO2 (TST90) [mins]	9.13 (32.30)	5.16 (15.16)	1.72 (8.84)	**0.076***	*0.157*†	0.224
Area under 90% SpO2 (CA90) [%*mins]	9.29 (62.20)	5.43 (23.99)	1.18 (12.94)	**0.07***	*0.129*†	0.233
SpO2 nadir [%]	80.00 (13.00)	79.50 (11.00)	83.00 (11.00)	0.02	*0.007*†	-0.177
Hypoxic burden [(%min)/h]	74.52 (76.11)	44.70 (42.46)	34.10 (31.29)	**0.214***	0.375	0.262

Variable	Group 1<br>Isolated REM-OSA	Group 2<br>HighTr	Group 3<br>LowTr	Effect size (η^2, Cliff's delta)<br>η^2 KW	 d^+ 1→2	 d^+ 2→3
**Demographics**						
Sex [F/M]	71 (55/16)	405 (228/177)	793 (426/367)	NA	NA	NA
Age [years]	66.00 (15.00)	67.00 (13.00)	69.00 (15.00)	0.007	*-0.007*†	-0.12
AHI [events/hour]	9.19 (5.05)	30.73 (20.97)	33.73 (28.78)	**0.14***	-0.939	*-0.085*†
**REM-OSA ratios**						
AHI ratio REM/NREM [-]	11.79 (11.39)	2.05 (2.00)	1.85 (1.68)	**0.133***	0.906	*0.077*†
Resp events ratio REM/NREM (Ev_{ratio}) [-]	2.78 (2.81)	0.65 (0.62)	0.33 (0.32)	**0.263***	0.814	0.487
TST ratio NREM/REM (T_{ratio}) [-]	0.25 (0.11)	0.30 (0.07)	0.18 (0.07)	**0.651***	-0.466	1.0
**Sleep features**						
Total Sleep Time (TST) [mins]	396.50 (87.50)	384.00 (83.50)	377.50 (86.50)	0.005	0.187	*0.041*†
Sleep Efficiency [%]	74.26 (11.24)	73.35 (14.40)	71.58 (14.19)	0.001	*0.073*†	*0.061*†
Epworth Sleepiness [ESS]	6.00 (6.00)	5.00 (6.00)	5.00 (5.00)	-0.001	*0.003*†	*0.052*†
ESS > 10 [#]	12	56	107	NA	NA	NA
Time in stage N1 / TST [%]	11.42 (9.51)	15.04 (9.90)	18.94 (14.44)	0.053	-0.262	-0.229
Time in stage N2 / TST [%]	53.68 (6.80)	48.78 (9.86)	53.22 (12.34)	0.054	0.409	-0.284
Time in stage N3 / TST [%]	11.88 (10.07)	10.52 (12.02)	10.69 (12.87)	-0.0	*0.127*†	*-0.005*†
Time in stage REM / TST [%]	20.03 (6.75)	22.87 (3.98)	15.29 (5.07)	**0.651***	-0.466	1.0
Time in stage REM [mins]	79.50 (32.75)	87.50 (24.00)	55.50 (22.50)	**0.443***	-0.212	0.823
Wake after Sleep Onset (WASO) [mins]	71.50 (74.25)	68.00 (70.50)	81.00 (73.50)	0.008	*0.021*†	-0.131
WASO longer than 5 minutes [mins]	47.00 (76.00)	40.50 (65.00)	50.00 (71.50)	0.002	*0.035*†	*-0.088*†
WASO - REM time [mins]	-12.50 (80.50)	-16.50 (76.00)	24.00 (82.50)	**0.122***	*0.088*†	-0.435
REM latency [mins]	82.50 (66.75)	81.00 (65.00)	105.00 (90.50)	0.033	*0.066*†	-0.233
Avg. duration of stage REM [mins]	10.44 (6.38)	10.57 (6.92)	7.35 (4.57)	**0.116***	*-0.052*†	0.415
Awakenings per hour [events/hour]	4.23 (1.66)	4.62 (2.29)	5.24 (2.84)	0.044	-0.228	-0.204
Arousals per hour [events/hour]	6.08 (4.95)	9.82 (8.55)	11.01 (9.74)	0.027	-0.354	*-0.092*†
**Position features**						
Time in position S / TST [%]	34.80 (58.10)	32.43 (49.08)	35.67 (51.77)	-0.002	*0.042*†	*-0.045*†
Time supine in REM / time in REM [%]	25.14 (62.29)	20.81 (56.03)	21.52 (62.39)	-0.001	*0.127*†	*-0.038*†
**OSA features**						
Percentage hypopnea events [%]	97.65 (5.80)	96.09 (7.62)	95.45 (10.51)	0.011	0.229	*0.067*†
Median duration of resp events Merged [s]	17.40 (4.77)	18.40 (4.95)	18.50 (5.35)	0.004	-0.196	*-0.023*†
**SpO2 features**						
Desaturations [#]	136.00 (104.50)	292.00 (206.00)	311.00 (224.00)	**0.069***	-0.649	*-0.067*†
Avg. desaturation duration [s]	32.68 (7.31)	33.74 (8.05)	33.81 (8.33)	-0.002	*-0.061*†	*-0.012*†
TST under 90% SpO2 (TST90) [mins]	0.42 (2.65)	4.45 (20.02)	6.08 (22.20)	0.036	-0.421	*-0.07*†
Area under 90% SpO2 (CA90) [%*mins]	0.32 (1.58)	4.09 (30.65)	6.29 (37.86)	0.038	-0.428	*-0.076*†
SpO2 nadir [%]	85.00 (9.50)	82.00 (12.00)	80.00 (12.00)	0.011	0.214	*0.074*†
Hypoxic burden [(%min)/h]	18.15 (19.30)	48.65 (53.35)	54.57 (59.12)	**0.082***	-0.686	*-0.075*†

Variable	REM-OSA groups			Isolated REM-OSA groups		
	REM-independent	LowEvr	HighEvr	Isolated	HighTr	LowTr
**Demographics**						
Sex [F/M]	270/367	91/39	348/154	55/16	228/177	426/367
Age [years]	0.05	0.08	-0.01	-0.13	-0.03	0.02
AHI [events/hour]	-0.11	0.16	-0.08	-0.16	-0.17	-0.25
**REM-OSA ratios**						
AHI ratio REM/NREM [-]	0.25	0.08	0.28	-0.01	**0.33***	**0.40***
Resp events ratio REM/NREM (Ev_{ratio}) [-]	0.19	0.02	0.22	-0.04	**0.34***	**0.35***
TST ratio NREM/REM (T_{ratio}) [-]	0.09	-0.11	-0.05	0.01	0.02	0.05
**Sleep features**						
Total Sleep Time (TST) [mins]	0.23	0.22	0.29	0.42	**0.31***	0.21
Sleep Efficiency [%]	0.07	0.18	0.07	0.07	0.11	0.07
Epworth Sleepiness [ESS]	-0.10	-0.33	-0.06	0.04	-0.09	-0.14
ESS > 10 [#]	NA	NA	NA	0.22	NA	NA
Time in stage N1 / TST [%]	**-0.36***	-0.35	**-0.40***	-0.32	**-0.50***	**-0.40***
Time in stage N2 / TST [%]	0.08	-0.15	-0.00	0.02	-0.00	0.12
Time in stage N3 / TST [%]	**0.36***	**0.47***	**0.38***	0.29	**0.51***	**0.36***
Time in stage REM / TST [%]	0.09	-0.11	-0.05	0.01	0.02	0.05
Time in stage REM [mins]	0.19	0.00	0.11	0.26	0.26	0.16
Wake after Sleep Onset (WASO) [mins]	-0.10	0.09	-0.06	-0.12	-0.10	-0.10
WASO longer than 5 minutes [mins]	-0.06	0.10	-0.05	-0.12	-0.06	-0.07
WASO - REM time [mins]	-0.17	0.07	-0.11	-0.19	-0.19	-0.15
REM latency [mins]	0.24	0.31	0.21	0.09	0.28	0.18
Avg. duration of stage REM [mins]	**0.30***	0.18	0.16	0.19	**0.33***	0.16
Awakenings per hour [events/hour]	**-0.32***	-0.22	-0.29	-0.32	**-0.43***	-0.29
Arousals per hour [events/hour]	-0.14	0.08	-0.06	0.10	-0.16	-0.21
**Position features**						
Time in position S / TST [%]	-0.02	0.02	0.23	-0.05	0.11	0.02
Time supine in REM / time in REM [%]	0.00	-0.08	0.24	0.03	0.13	0.06
**OSA features**						
Percentage hypopnea events [%]	0.15	0.15	-0.03	-0.10	0.07	0.21
Median duration of resp events Merged [s]	-0.24	-0.15	-0.19	-0.16	-0.20	-0.29
**SpO2 features**						
Desaturations [#]	-0.03	0.15	0.05	0.20	-0.11	-0.11
Avg. desaturation duration [s]	-0.23	-0.17	-0.20	-0.15	-0.19	-0.25
TST under 90% SpO2 (TST90) [mins]	-0.05	0.01	0.03	0.08	-0.04	-0.11
Area under 90% SpO2 (CA90) [%*mins]	-0.05	0.04	0.06	0.10	-0.03	-0.08
SpO2 nadir [%]	-0.03	-0.14	-0.09	0.21	0.00	-0.06
Hypoxic burden [(%min)/h]	-0.19	-0.02	-0.09	0.00	-0.23	-0.26

S1.3 dataset SOMNIA

Variable	Group 1 REM-independent	Group 2 LowEvr	Group 3 HighEvr	Effect size (η^2, Cliff's delta) η^2 KW	d$^+$ 1→2	d$^+$ 2→3
**Demographics**						
Sex [F/M]	215 (48/167)	22 (8/14)	69 (35/34)	NA	NA	NA
Age [years]	55.00 (15.00)	49.50 (18.00)	53.00 (13.00)	-0.002	*0.131*†	*-0.026*†
BMI [kg/m^2]	27.67 (4.84)	28.72 (7.35)	28.06 (7.26)	-0.0	*-0.263*†	*0.205*†
AHI [events/hour]	23.73 (24.77)	24.99 (16.61)	9.04 (7.62)	**0.24***	*-0.072*†	0.714
**REM-OSA ratios**						
AHI ratio REM/NREM [-]	0.88 (0.75)	2.20 (0.66)	4.44 (4.20)	**0.626***	-1.0	*-0.74*†
Resp events ratio REM/NREM (Ev_{ratio}) [-]	0.20 (0.21)	0.38 (0.11)	1.17 (1.11)	**0.557***	-0.645	-1.0
TST ratio NREM/REM (T_{ratio}) [-]	0.23 (0.09)	0.15 (0.07)	0.25 (0.10)	**0.154***	0.677	-0.949
**Sleep features**						
Total Sleep Time (TST) [mins]	412.00 (78.00)	388.75 (135.00)	415.50 (85.50)	-0.006	*0.187*†	*-0.194*†
Sleep Efficiency [%]	81.65 (13.41)	75.25 (15.14)	84.64 (12.56)	0.016	0.319	-0.424
Epworth Sleepiness [ESS]	11.00 (10.00)	13.00 (7.50)	12.00 (8.00)	-0.018	*-0.105*†	*0.059*†
ESS > 10 [#]	72	10	23	NA	NA	NA
Time in stage N1 / TST [%]	16.67 (11.56)	17.47 (10.53)	10.92 (6.75)	**0.092***	*-0.101*†	0.578
Time in stage N2 / TST [%]	51.95 (11.40)	53.33 (5.72)	52.50 (9.07)	-0.004	*-0.141*†	*0.054*†
Time in stage N3 / TST [%]	12.01 (9.36)	11.76 (8.88)	13.82 (10.06)	-0.006	*0.115*†	*-0.024*†
Time in stage REM / TST [%]	18.43 (6.26)	12.73 (5.07)	20.20 (5.97)	**0.154***	0.678	-0.95
Time in stage REM [mins]	74.50 (33.00)	44.75 (13.75)	82.50 (27.50)	**0.126***	0.656	-0.872
Wake after Sleep Onset (WASO) [mins]	70.00 (64.25)	99.75 (64.50)	58.50 (71.00)	-0.001	*-0.11*†	*0.217*†
WASO longer than 5 minutes [mins]	41.00 (56.25)	59.75 (48.62)	32.00 (58.50)	-0.005	*-0.143*†	*0.212*†
WASO - REM time [mins]	-4.50 (77.50)	41.25 (87.25)	-28.00 (87.00)	0.04	-0.345	0.542
REM latency [mins]	100.00 (90.00)	146.75 (133.75)	92.50 (37.50)	0.036	-0.393	0.577
Avg. duration of stage REM [mins]	10.62 (5.91)	5.78 (3.32)	9.31 (4.36)	**0.069***	0.634	-0.603
Awakenings per hour [events/hour]	4.38 (2.40)	4.44 (2.51)	3.87 (1.86)	0.019	*-0.06*†	*0.298*†
Arousals per hour [events/hour]	16.63 (11.42)	18.38 (7.62)	11.71 (6.09)	**0.113***	*-0.113*†	0.619
**Position features**						
Time in position S / TST [%]	29.03 (35.66)	39.03 (57.39)	37.54 (45.33)	-0.011	*-0.115*†	*0.082*†
Time supine in REM / time in REM [%]	19.31 (46.49)	53.57 (70.23)	34.85 (63.97)	0.014	*-0.28*†	*0.147*†
**OSA features**						
Percentage hypopnea events [%]	92.16 (23.32)	92.18 (23.02)	95.83 (14.71)	0.022	*-0.024*†	*-0.248*†
Median duration of resp events Merged [s]	20.95 (6.03)	20.55 (6.58)	20.76 (5.06)	-0.012	*0.069*†	*-0.055*†
**SpO2 features**						
Desaturations [#]	110.00 (157.00)	113.00 (99.50)	56.00 (60.00)	**0.08***	*-0.047*†	0.562
Avg. desaturation duration [s]	32.53 (10.17)	29.57 (10.85)	30.00 (9.98)	0.005	*0.232*†	*-0.118*†
TST under 90% SpO2 (TST90) [mins]	1.74 (10.54)	1.95 (7.94)	0.63 (4.95)	-0.002	*-0.09*†	*0.26*†
Area under 90% SpO2 (CA90) [%*mins]	1.93 (17.72)	4.22 (16.26)	0.60 (9.29)	-0.003	*-0.133*†	*0.258*†
SpO2 nadir [%]	84.69 (8.81)	83.16 (5.88)	85.79 (7.77)	-0.002	*0.178*†	*-0.265*†
Hypoxic burden [(%min)/h]	21.94 (40.13)	27.18 (27.11)	7.97 (17.33)	**0.07***	*-0.053*†	0.523

Variable	Group 1<br>Isolated REM-OSA	Group 2<br>HighTr	Group 3<br>LowTr	Effect size (η^2, Cliff's delta)<br>η^2 KW	 d^+ 1→2	 d^+ 2→3
**Demographics**						
Sex [F/M]	32 (17/15)	99 (21/78)	175 (53/122)	NA	NA	NA
Age [years]	50.50 (13.50)	53.00 (15.00)	55.00 (14.50)	0.013	*-0.204*†	*-0.088*†
BMI [kg/m^2]	28.23 (7.79)	26.82 (4.76)	28.07 (5.51)	0.001	*0.109*†	*-0.15*†
AHI [events/hour]	6.65 (3.12)	21.36 (19.23)	22.31 (21.95)	**0.213***	-0.92	*-0.009*†
**REM-OSA ratios**						
AHI ratio REM/NREM [-]	7.34 (8.08)	1.15 (0.92)	1.12 (1.30)	**0.246***	0.956	*0.039*†
Resp events ratio REM/NREM (Ev_{ratio}) [-]	1.90 (1.34)	0.35 (0.32)	0.20 (0.24)	**0.321***	0.934	0.371
TST ratio NREM/REM (T_{ratio}) [-]	0.25 (0.10)	0.29 (0.07)	0.19 (0.07)	**0.656***	-0.363	1.0
**Sleep features**						
Total Sleep Time (TST) [mins]	410.50 (95.25)	432.50 (80.75)	399.50 (87.00)	0.024	*-0.186*†	0.241
Sleep Efficiency [%]	83.20 (13.20)	85.32 (13.14)	79.96 (14.89)	0.031	*-0.099*†	0.262
Epworth Sleepiness [ESS]	9.50 (8.25)	13.00 (9.00)	11.00 (11.00)	-0.011	*-0.074*†	*0.126*†
ESS > 10 [#]	8	39	58	NA	NA	NA
Time in stage N1 / TST [%]	10.80 (5.53)	15.61 (8.73)	16.00 (11.94)	0.043	-0.433	*-0.052*†
Time in stage N2 / TST [%]	52.77 (9.56)	48.37 (8.51)	54.57 (9.56)	**0.11***	0.371	-0.439
Time in stage N3 / TST [%]	14.08 (6.72)	11.43 (8.49)	12.32 (10.22)	-0.001	*0.232*†	*-0.08*†
Time in stage REM / TST [%]	20.28 (5.90)	22.64 (4.06)	16.22 (4.73)	**0.656***	-0.364	1.0
Time in stage REM [mins]	79.50 (30.75)	96.00 (20.25)	62.50 (25.75)	**0.515***	-0.366	0.9
Wake after Sleep Onset (WASO) [mins]	56.00 (73.50)	56.50 (55.00)	74.50 (64.00)	0.024	*-0.025*†	-0.236
WASO longer than 5 minutes [mins]	36.00 (68.38)	27.50 (44.50)	46.50 (62.50)	0.019	*0.086*†	-0.227
WASO - REM time [mins]	-32.25 (98.88)	-41.00 (68.00)	16.50 (76.00)	**0.182***	*0.156*†	-0.548
REM latency [mins]	94.75 (33.38)	80.50 (46.25)	109.50 (93.50)	0.053	*0.12*†	-0.317
Avg. duration of stage REM [mins]	9.71 (4.15)	10.80 (6.20)	9.08 (5.56)	0.033	*-0.162*†	0.27
Awakenings per hour [events/hour]	3.75 (1.52)	4.14 (2.04)	4.45 (2.46)	0.016	*-0.218*†	*-0.102*†
Arousals per hour [events/hour]	10.39 (4.53)	15.73 (8.37)	16.82 (11.87)	**0.075***	-0.546	*-0.069*†
**Position features**						
Time in position S / TST [%]	31.29 (40.92)	35.52 (43.37)	28.45 (37.58)	-0.007	*-0.07*†	*0.105*†
Time supine in REM / time in REM [%]	26.20 (63.75)	26.82 (54.15)	21.55 (53.82)	-0.012	*-0.026*†	*0.039*†
**OSA features**						
Percentage hypopnea events [%]	97.84 (7.63)	94.37 (17.21)	91.30 (23.88)	0.031	0.341	*0.094*†
Median duration of resp events Merged [s]	20.19 (4.88)	21.50 (6.19)	20.95 (5.91)	-0.007	*-0.158*†	*0.023*†
**SpO2 features**						
Desaturations [#]	34.00 (37.50)	103.00 (132.50)	106.00 (139.00)	**0.078***	-0.494	*-0.07*†
Avg. desaturation duration [s]	28.21 (7.90)	33.19 (10.76)	32.26 (9.98)	0.015	-0.306	*0.001*†
TST under 90% SpO2 (TST90) [mins]	0.27 (5.11)	0.97 (8.58)	1.78 (10.31)	0.002	*-0.136*†	*-0.088*†
Area under 90% SpO2 (CA90) [%*mins]	0.29 (10.31)	1.76 (9.54)	1.93 (18.24)	-0.003	*-0.1*†	*-0.073*†
SpO2 nadir [%]	85.81 (7.92)	85.32 (9.71)	84.20 (7.66)	-0.005	*0.03*†	*0.11*†
Hypoxic burden [(%min)/h]	5.18 (5.38)	21.29 (33.98)	21.81 (38.96)	**0.081***	-0.493	*-0.086*†

Variable	REM-OSA groups			Isolated REM-OSA groups		
	REM-independent	LowEvr	HighEvr	Isolated	HighTr	LowTr
**Demographics**						
Sex [F/M]	48/167	8/14	35/34	17/15	21/78	53/122
Age [years]	0.04	0.25	0.28	0.33	0.07	0.09
AHI [events/hour]	-0.21	0.48	0.02	0.02	-0.39	-0.10
BMI [kg/m^2]	-0.05	0.32	-0.02	-0.00	-0.19	0.07
**REM-OSA ratios**						
AHI ratio REM/NREM [-]	0.05	0.05	0.14	0.19	0.37	0.17
Resp events ratio REM/NREM (Ev_{ratio}) [-]	0.00	-0.09	0.07	0.10	0.36	0.17
TST ratio NREM/REM (T_{ratio}) [-]	-0.12	-0.04	-0.21	-0.29	0.06	0.05
**Sleep features**						
Total Sleep Time (TST) [mins]	-0.04	0.04	-0.06	-0.25	-0.09	0.07
Sleep Efficiency [%]	-0.07	0.04	-0.04	-0.04	-0.14	0.02
Epworth Sleepiness [ESS]	-0.09	0.00	0.10	0.13	-0.15	0.02
ESS > 10 [#]	-0.13	-0.23	0.12	0.13	-0.04	-0.06
Time in stage N1 / TST [%]	-0.29	-0.02	-0.26	-0.29	-0.33	-0.30
Time in stage N2 / TST [%]	0.16	0.21	0.20	0.33	0.02	0.17
Time in stage N3 / TST [%]	0.21	0.00	0.22	0.18	0.31	0.15
Time in stage REM / TST [%]	-0.12	-0.05	-0.21	-0.29	0.06	0.05
Time in stage REM [mins]	-0.09	0.16	-0.24	-0.43	-0.07	0.08
Wake after Sleep Onset (WASO) [mins]	0.02	0.12	0.04	0.02	0.04	-0.03
WASO longer than 5 minutes [mins]	0.08	0.21	0.03	0.01	0.11	0.02
WASO - REM time [mins]	0.05	0.12	0.16	0.22	0.08	-0.06
REM latency [mins]	0.20	0.54	0.33	0.48	0.25	0.12
Avg. duration of stage REM [mins]	0.22	-0.24	-0.04	0.18	0.06	0.09
Awakenings per hour [events/hour]	-0.07	0.14	0.06	0.14	-0.02	-0.09
Arousals per hour [events/hour]	-0.12	0.36	0.36	0.46	0.02	-0.11
**Position features**						
Time in position S / TST [%]	0.19	0.44	0.23	0.29	0.22	0.23
Time supine in REM / time in REM [%]	0.16	0.47	0.23	0.33	0.41	0.16
**OSA features**						
Percentage hypopnea events [%]	0.29	0.27	-0.32	-0.29	0.07	0.25
Median duration of resp events Merged [s]	-0.11	-0.01	-0.19	-0.25	0.07	-0.14
**SpO2 features**						
Desaturations [#]	-0.19	0.48	-0.13	-0.31	-0.34	-0.07
Avg. desaturation duration [s]	-0.09	0.21	-0.15	-0.39	0.09	-0.10
TST under 90% SpO2 (TST90) [mins]	-0.12	0.43	-0.01	-0.01	-0.27	0.02
Area under 90% SpO2 (CA90) [%*mins]	-0.11	0.39	-0.04	0.00	-0.27	0.01
SpO2 nadir [%]	0.00	-0.20	-0.07	-0.18	0.05	0.01
Hypoxic burden [(%min)/h]	-0.18	0.41	-0.15	-0.32	-0.23	-0.10
